# The Finally Rewarding Search for A Cytotoxic Isosteviol Derivative

**DOI:** 10.3390/molecules28134951

**Published:** 2023-06-23

**Authors:** Julia Heisig, Niels V. Heise, Sophie Hoenke, Dieter Ströhl, René Csuk

**Affiliations:** Organic Chemistry, Martin-Luther University Halle-Wittenberg, Kurt-Mothes, Str. 2, D-06120 Halle (Saale), Germany; julia.heisig@student.uni-halle.de (J.H.); niels.heise@chemie.uni-halle.de (N.V.H.); sophie.hoenke@chemie.uni-halle.de (S.H.); dieter.stroehl@chemie.uni-halle.de (D.S.)

**Keywords:** stevioside, isosteviol, rhodamine conjugates, cytotoxicity

## Abstract

Acid hydrolysis of stevioside resulted in a 63% yield of isosteviol (**1**), which served as a starting material for the preparation of numerous amides. These compounds were tested for cytotoxic activity, employing a panel of human tumor cell lines, and almost all amides were found to be non-cytotoxic. Only the combination of isosteviol, a (homo)-piperazinyl spacer and rhodamine B or rhodamine 101 unit proved to be particularly suitable. These spacered rhodamine conjugates exhibited cytotoxic activity in the sub-micromolar concentration range. In this regard, the homopiperazinyl-spacered derivatives were found to be better than those compounds with piperazinyl spacers, and rhodamine 101 conjugates were more cytotoxic than rhodamine B hybrids.

## 1. Introduction

**Scheme 1:**
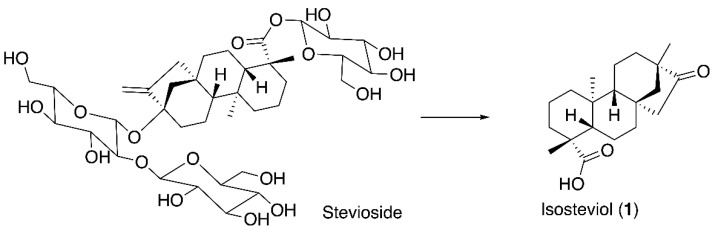
Hydrolysis (methanolic HCl, ∆, 2 h, 63%) of stevioside to isosteviol (**1**).

At least since the introduction and approval of stevioside [1,2,3,4,5] as a sweetener and substitute for sugar, increased attention has been paid to its aglycone steviol and its rearrangement product isosteviol (**1**, Scheme 1) [3,4]. Stevioside (Scheme 1) is now present in numerous food and consumer goods in daily life, as it is a non-caloric sugar substitute with a sweetening power of 250–300 times sweeter than sucrose. In addition, numerous therapeutic benefits are attributed to it, including antihyperglycemic, antihypertensive, anti-inflammatory, antitumor, and immunomodulatory properties [6,7,8,9,10,11]. Alkaline as well as enzymatic hydrolysis of stevioside leads to steviol, while acid hydrolysis leads to isosteviol (**1**) [12,13,14,15,16,17]. Isosteviol is a diterpene of the *ent*-beyerane = stachane type, i.e., a 13-methyl-17-norkaurane; it is obtained biosynthetically from the cyclization of a pimarane cation intermediate without subsequent rearrangement.

Isosteviol has been reported to be a suppressor of human DNA topoisomerase II [18] and of mammalian DNA polymerases, thus explaining its antitumor activity [19,20]. Parent stevioside was reported to have minor cytotoxic effects on several human cancer cell lines, thereby inducing apoptosis and cell cycle arrest in the G_2_/M phase [21,22,23,24,25,26,27,28,29,30,31,32,33,34,35,36,37,38]. Since previous work also indicated an effect on mitochondrial permeability transition [35], it was natural to transfer our own previous findings on pentacyclic triterpenes to the diterpene isosteviol. Of particular interest were benzyl and pyridine-amides, (iso)-quinoline amides, and especially (homo)-piperazinyl-spacered rhodamine B and rhodamine 101 conjugates [39,40,41,42,43,44,45,46,47,48,49,50,51,52,53,54,55,56,57,58].

Quinoline (and isoquinoline) derivatives of pentacyclic triterpenes had shown high cytotoxicity with partly excellent tumor cell/non-tumor cell selectivity [56], while several benzylamides were of good cytotoxicity but also excellent selectivity [43,46,47,48,53,55,58,59,60]; rhodamine B conjugates of triterpenoic acid held excellent cytotoxic activity; for some derivatives even sub-nanomolar cytotoxicity has been reported [51].

Previous results from our labs revealed a dependance on the type of spacer between the triterpene and the cationic rhodamine moiety. Thereby, piperazinyl and homopiperazinyl spacer proved especially useful; hence, these two spacers were included in this study [45,46,51,55,57].

Many solid tumor cells hold a trans-membrane potential exceeding that of “normal” cells, thus allowing a selective accumulation of lipophilic delocalized cations in the tumor cell mitochondria [61,62,63,64,65,66,67,68]. Our previous studies on these cationic hybrids were limited to pentacyclic triterpenoid hybrids; the first results obtained for diterpenoid abietylamine indicated some potential also for non-pentacyclic triterpenoids [69,70,71,72]. It seems that, depending on both the kind of terpene as well as on the chosen cation, different modes of action are involved. While for triterpenoid-safirinium compounds [73], as well as for an aza-BODIPY [74], an accumulation in the endoplasmic reticulum was observed; several rhodamine conjugates interact in the mitochondria [46], and even an almost complete shut-down of the mitochondrial ATP synthesis has been observed for an Asiatic acid-derived hybrid [51].

## 2. Results

Stevioside (obtained from different local vendors) was hydrolyzed in methanolic hydrochloric acid, and isosteviol (**1**) was obtained in a 63% isolated yield. Due to some ambiguity in the assignment of its ^1^H and ^13^C NMR spectra [75,76,77], a complete analysis was undertaken. However, it quickly became apparent that the usual 1D and 2D NMR experiments (e.g., gHSQC and gHMBC) did not allow a complete and accurate assignment of all signals, since even in these spectra, some signals overlapped very strongly. In addition, data from the literature contradicted each other with respect to the assignment of the quaternary carbons. To solve this problem, further NMR experiments were carried out, with which it was possible to directly separate the connectivity in the carbon framework. Two methods are suitable for this purpose: On the one hand, this is the classical INADEQUATE method [78,79,80,81], which requires larger substance amounts but also long measurement times due to the low sensitivity, or, on the other hand, the 1,1-ADEQUATE experiment [82,83,84]. In contrast to the INADEQUATE experiment, which is based on a correlation of the ^1^J coupling between two vicinal ^13^C nuclei, the 1,1-ADEQUATE experiment uses an INEPT transfer between ^1^H and ^13^C adjacent nuclei and the subsequent formation of double quantum coherence, thereby circumventing the problem of the low natural abundance of ^13^C nuclei.

**Figure 1:**
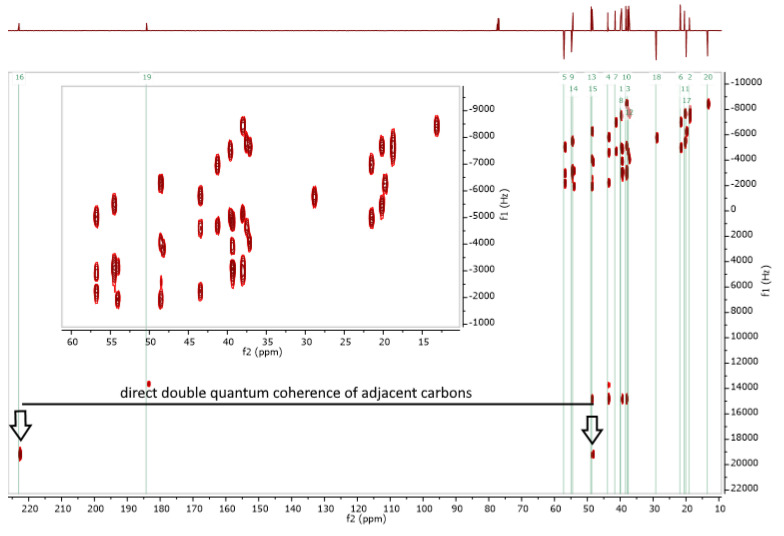
INADEQUATE spectrum of **1** in CDCl_3_; total acquisition time: 80 h.

**Figure 2:**
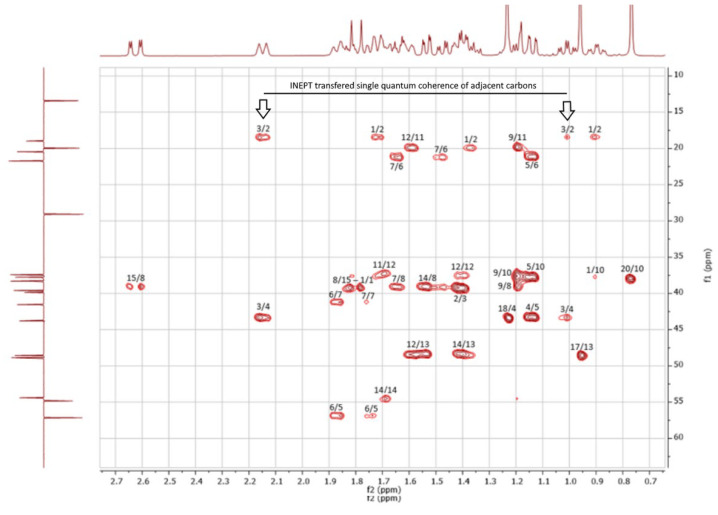
1,1-ADEQATE spectrum of **1** in CDCl_3_; total acquisition time: 20 h.

So far, no INADEQUATE or 1,1-ADEQUATE spectra have been reported in the literature specifically for isosteviol. Due to the very good solubility of isosteviol in chloroform (520 mg in 0.7 mL) and yet sufficiently low viscosity at the measuring temperature used (27 °C), it was possible to perform both experiments and compare the results. While the INADEQUATE experiment required a measuring time of 80 h, 20 h was sufficient for the ADEQUATE experiment. The results are shown in Figure 1 and Figure 2 (in the ADEQUATE experiment, the chemical shift is shown in ppm instead of the double quantum coherence frequency, for better comparability). It was shown that both methods are very well suited for an exact and doubtless assignment. However, it is also evident that the INADEQUATE spectrum is somewhat easier to interpret, since no overlapping proton signals have to be taken into account. Both NMR experiments, however, allow a doubtless and reliable assignment of all signals.

**Scheme 2:**
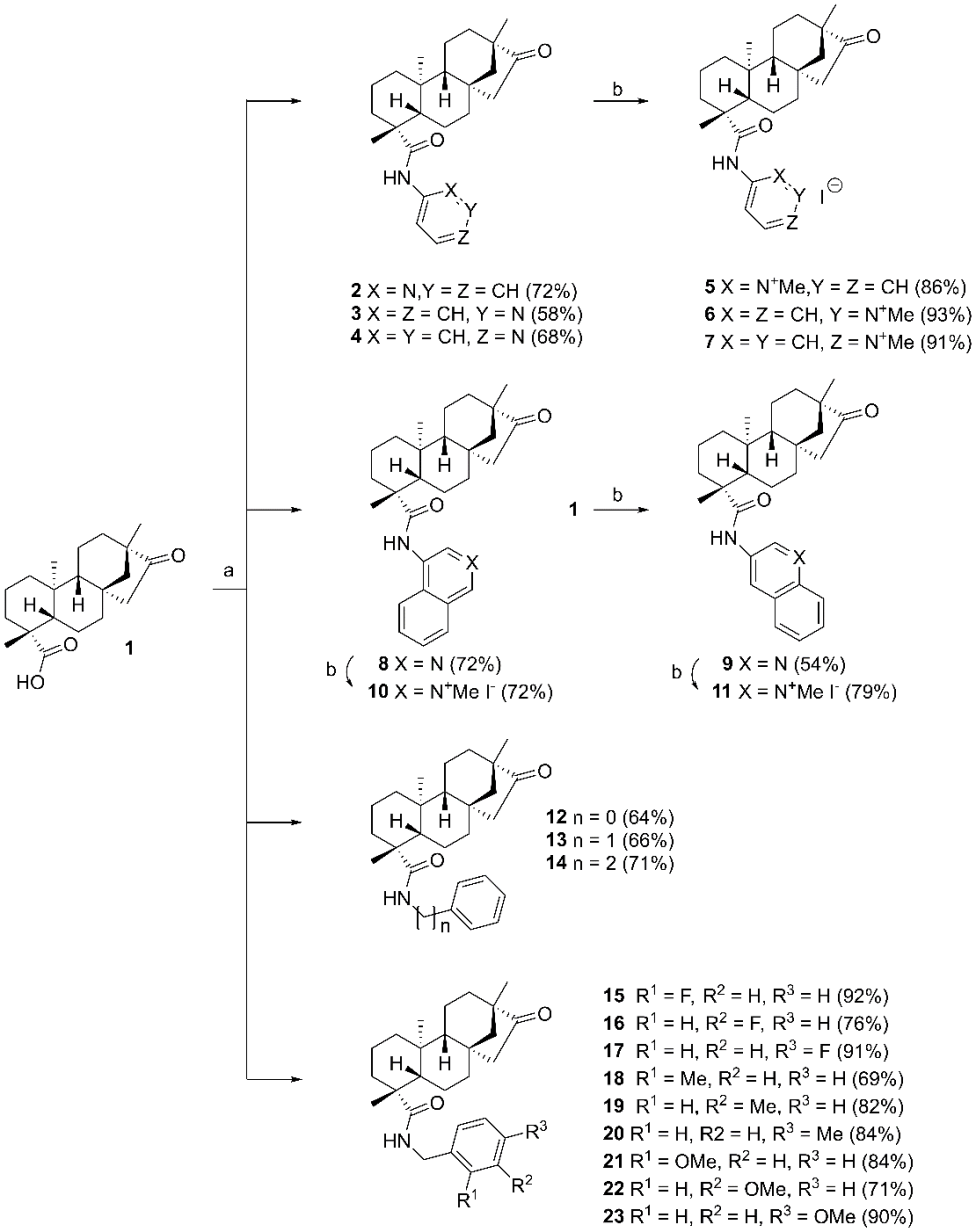
Synthesis of isosteviol derived pyridinyl-, (iso)-quinolinyl- and benzylamides. Reactions and conditions: (a) (COCl)_2_, DCM, DMF (cat.), 1 h, 23 °C, then amine in DCM, 23 °C, 1 h; (b) CH_3_I, DCM, 23 °C, 2 h.

**Scheme 3:**
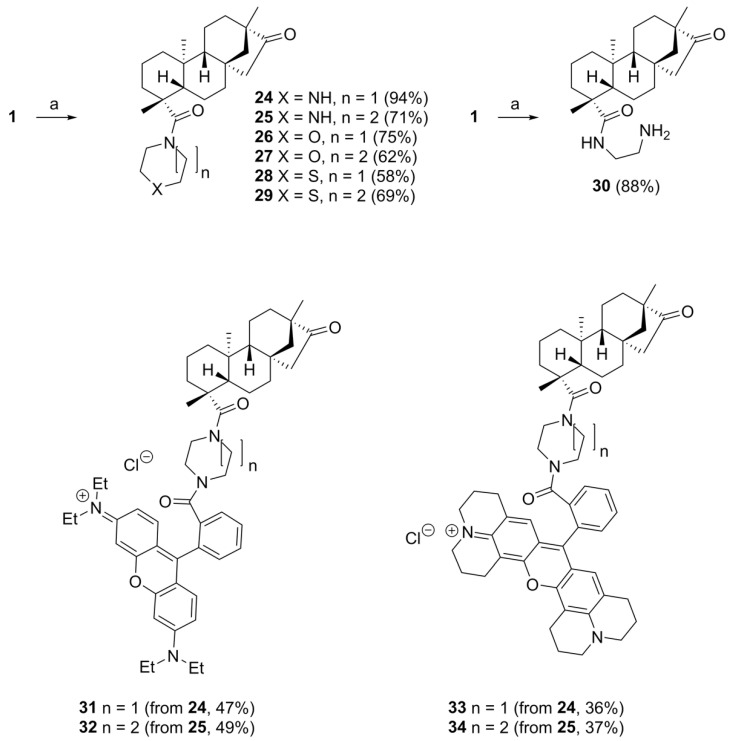
Synthesis and structure of amides **24**–**30** as well as rhodamine B (**31**, **32**) and rhodamine 101 conjugates **33** and **34**. Reactions and conditions: (a) (COCl)_2_, DCM, DMF (cat.), 1 h, 23 °C, then amine in DCM, 23 °C, 1 h.

Compound 1 was transformed in situ with oxalyl chloride into the corresponding acid chloride followed by the addition of the corresponding amine (Scheme 2). The pyridine amides **2**–**4**, the quinoline amides **8** and **9,** the amides **12**–**23**, the (homo)-piperazinyl amides **24** and **25** (Scheme 3), the (homo)-morpholinyl amides **26** and **27**, and the (homo)-thiomorpholinyl amides **28** and **29** were obtained; compound **30** was synthesized from **1** and ethylene diamine in 88% yield. Rhodamine B or rhodamine 101 was activated with oxalyl chloride in the same manner, followed by the reaction with either **26** and **27** to yield **31** and **32** (from rhodamine B) or **33** and **34** (from rhodamine 101), respectively. We refrained from using **30** as a starting material to prepare the corresponding rhodamine conjugates, since it has since become apparent that these conjugates exist preferentially in a non-cationic but neutral spirocyclic form. These electrically neutral molecules proved to be hardly cytotoxic, since they obviously cannot interact with membranes.

For comparison, quaternization was performed with iodomethane, and this provided **5**–**7** as well as **10** and **11**, respectively.

Isosteviol (**1**) and all derivatives were subjected to sulforhodamine B (SRB) assays employing a panel of human tumor cell lines and non-malignant murine fibroblasts NIH 3T3 and HEK293 cells for comparison. The results from these assays are compiled in Table 1.

The SRB assays showed neither the parent compound isosteviol (**1**) nor almost none of the amides to hold any cytotoxic effect on the human tumor cell lines; they were also non-cytotoxic for the non-malignant cell lines NIH 3T3 and HEK293. The (homo)-piperazinyl amides **24** and **25**, however, showed slight cytotoxic effect, with the homopiperazinyl amide **25** performing slightly better than the piperazinyl-spacered compound **24**. A significant improvement was made with the (homo)-piperazinyl rhodamine-B-spacered compounds **31** and **32**, and the rhodamine 101 hybrids **33** and **34** were even more cytotoxic than the rhodamine B analogs **31** and **32**. The selectivity to distinguish between malignant and non-malignant cell lines, however, was low.

These results emphasize once again that for high cytotoxic activity of terpene/rhodamine hybrids, the interplay between the selected terpene, spacer and lipophilic cation is crucial. If the cationic part is not lipophilic enough (as in compounds **6**–**7**, **10** and **11**), no cytotoxic activity is obtained. The values obtained for isosteviol derivatives are basically much smaller than those previously obtained for pentacyclic triterpenes. On the other hand, a pronounced cytotoxicity can be achieved also for non-cytotoxic isosteviol if an appropriate rhodamine residue is added to the terpenoid backbone via a suitable spacer. Once again, the homopiperazinyl spacer proves to be superior to the piperazinyl spacer.

## 3. Experiment

NMR spectra were recorded using the Varian spectrometers (Darmstadt, Germany) DD2 and VNMRS (400 and 500 MHz, respectively). MS spectra were taken on a Advion expressionL CMS mass spectrometer (Ithaca, USA; positive ion polarity mode, solvent: methanol, solvent flow: 0.2 mL/min, spray voltage: 5.17 kV, source voltage: 77 V, APCI corona discharge: 4.2 μA, capillary temperature: 250 °C, capillary voltage: 180 V, sheath gas: N2). Thin-layer chromatography was performed on pre-coated silica gel plates supplied by Macherey-Nagel (Düren, Germany). IR spectra were recorded on a Spectrum 1000 FT-IR-spectrometer from Perkin Elmer (Rodgau, Germany). The UV/Vis-spectra were recorded on a Lambda 14 spectrometer from Perkin Elmer (Rodgau, Germany); optical rotations were measured using a JASCO-P2000 instrument (JASCO Germany GmbH, Pfungstadt, Germany). The melting points were determined using the Leica hot stage microscope Galen III (Leica Biosystems, Nussloch, Germany) and are uncorrected. The solvents were dried according to usual procedures. Microanalyses were performed with an Elementar Vario EL (CHNS) instrument (Elementar Analysensysteme GmbH, Elementar-Straße 1, D-63505 Langenselbold, Germany). All dry solvents were distilled over respective drying agents, except for DMF, which was distilled and stored under argon and molecular sieve. Reactions using air- or moisture-sensitive reagents were carried out under argon atmosphere in dried glassware. Triethylamine was stored over potassium hydroxide. Biological assays were performed as previously reported, employing cell lines obtained from the Department of Oncology [Martin-Luther-University Halle Wittenberg; they were bought from ATCC: malignant: A 375, HT29, MCF7 and A2780; non-malignant: NIH 3T3]. Rhodamine B and stevioside were obtained from local vendors and used as received.

For the SRB assay: cells were seeded into 96-well plates on day zero at appropriate cell densities to prevent confluence of the cells during the period of the experiment. After 24 h, the cells were treated with different concentrations (1, 3, 7, 12, 20 and 30 μM), but the final concentration of DMSO/DMF never exceeded 0.5%, which was non-toxic to the cells. After 72 h of treatment, the supernatant media from the 96-well plates were discarded, and then the cells were fixed with 10% trichloroacetic acid and allowed to rest at 4 °C. After 24 h of fixation, the cells were washed in a strip washer and then dyed with SRB solution (200 μL, 10 mM) for 20 min. Then the plates were washed four times with 1% acetic acid to remove the excess dye and allowed to air-dry overnight. Tris base solution (200 μL, 10 mM) was added to each well. The absorbance was measured with a 96-well plate reader from Tecan Spectra.

### 3.1. General Procedure for the Synthesis of Amides (GPA)

A solution of **1** (1 equiv.) in dry DCM (10 mL) was treated with oxalyl chloride (4 equiv.) and DMF (catal.) for 1 h. The volatiles were evaporated under reduced pressure. To a solution of the residue in dry DCM (10 mL) the corresponding amine (3 equiv.) was added, and the mixture was stirred at room temperature for 1 h. The usual aqueous workup, followed by chromatography, gave amides.

### 3.2. General Procedure for the Quaternization (GPB)

To a solution of **2**–**4**, **8,** or **9** in dry DCM (3 mL), iodomethane (3 mL, 0.05 mmol) was added, and the mixture was stirred at room temperature for 2 h. The volatiles were evaporated under reduced pressure, and the residue was subjected to chromatography to afford **5**–**6**, **10**, and **11**.

### 3.3. General Procedure for the Synthesis of Rhodamine Conjugates (GPC)

The respective rhodamine was dissolved in dry dichloromethane (10 mL) and mixed with oxalyl chloride (4 eq.) and catalytic amounts of DMF. Following the conditions of GPA as described above, the residue was dissolved in dry DCM (10 mL) and compounds **22** or **23** (3 eq.) were added. Stirring at room temperature was continued for 1 h. The usual aq. work-up followed by chromatography furnished the conjugates **31**–**34**.

### 3.4. 16-Oxostachan-18-oic Acid (1, Isosteviol)

A suspension of stevioside (43.0 g, from different local vendors) in MeOH (250 mL) and conc. aq. HCl (43 mL) was heated under reflux for 2 h; stirring at room temperature was continued overnight. Precipitation with water (600 mL) gave a solid that was re-crystallized from EtOH (150 mL), and **1** (10.8 g, 63%) was obtained as a colorless solid; R_f_ = 0.69 (SiO_2_, CHCl_3_/MeOH, 9:1); m.p. 230 °C (lit.: [85] 228–230 °C); ${\left[\alpha \right]}_{D}^{20}$ = −84.45° (*c* = 0.164, CHCl_3_); IR (ATR): ν = 2959*w*, 2922*w*, 2851*w*, 1736*m*, 1690*m*, 1455*w*, 1264*w* cm^−1^; ^1^H NMR (500 MHz, CDCl_3_): δ = 2.62 (*dd*, *J* = 18.6, 3.7 Hz, 1H, 15-H), 2.15 (*dt*, *J* = 13.1, 4.0 Hz, 1H, 3-H), 1.91 − 1.79 (*m*, 3H, 2-H, 6-H, 15-H), 1.79 − 1.67 (*m*, 3H, 1-H, 6-H, 11-H), 1.64 (*dt*, *J* = 13.3, 3.1 Hz, 2H, 7-H), 1.62 − 1.57 (*m*, 1H, 12-H), 1.53 (*dd*, *J* = 11.6, 2.7 Hz, 1H, 14-H), 1.48 (*dd*, *J* = 13.6, 4.0 Hz, 1H, 7-H), 1.44 − 1.33 (*m*, 3H, 2-H, 12-H, 14-H), 1.23 (*s*, 3H, 20-H), 1.21 − 1.17 (*m*, 1H, 9-H), 1.14 (*dd*, *J* = 12.1, 2.3 Hz, 1H, 5-H), 1.01 (*td*, *J* = 13.6, 4.2 Hz, 1H, 3-H), 0.96 (*s*, 3H, 17-H), 0.90 (*td*, *J* = 13.3, 4.3 Hz, 1H, 1-H), 0.77 (*s*, 3H, 19-H) ppm; ^13^C NMR (126 MHz, CDCl_3_): δ = 222.94 (C-16), 184.16 (C-18), 57.13 (C-5), 54.84 (C-9), 54.38 (C-14), 48.85 (C-13), 48.55 (C-15), 43.79 (C-4), 41.55 (C-7), 39.86 (C-1), 39.59 (C-8), 38.30 (C-10), 37.75 (C-3), 37.42 (C-12), 29.07 (C-20), 21.73 (C-6), 20.45 (C-11), 19.95 (C-17), 18.97 (C-2), 13.42 (C-19) ppm; MS (ESI, MeOH/CHCl_3_, 4:1): *m/z* (%) = 317 (100%, [M-H]^−^).

### 3.5. 16-Oxo-N-pyridin-2-yl-stachan-18-amide (2)

Following GPA from **1** (300 mg, 0.94 mmol), oxalyl chloride (0.4 mL (4.7 mmol), 2-aminopyridine (354 mg, 3.76 mmol), NEt_3_ (0.7 mL, 5.0 mmol), and chromatography (SiO_2_, CHCl_3_/MeOH, 9:1), **2** (267 mg, 72%) was obtained as a colorless solid; R_f_ = 0.16 (SiO_2_, hexanes/ethyl acetate, 8:2); m.p. 185.5 °C; ${\left[\alpha \right]}_{D}^{20}$ = −73.47° (*c* = 0.034, MeOH); UV-Vis (MeOH): λ_max_ (log ε) = 278.52 nm (0.37); IR (ATR) (ATR): ν = 3442*w*, 2926*m*, 2847*m*, 1735*s*, 1683*m*, 1592*w*, 1576*m*, 1504*m*, 1453*w*, 1427*s*, 1295*m*, 1253*w*, 1210*w*, 1177*w*, 1147*m*, 1131*w*, 1109*w*, 1090*w*, 1050*w*, 1016*w*, 929*w*, 868*w*, 778*m*, 753*m*, 695*w*, 666*w*, 611*w*, 518*w*, 410*w* cm^−1^; ^1^H NMR (500 MHz, CDCl_3_): δ = 8.50 (*s*, 1H, N-H), 8.29 − 8.22 (*m*, 2H, 22-H, 25-H), 7.77 (*ddd*, *J* = 8.7, 7.3, 1.9 Hz, 1H, 23-H), 7.08 (*ddd*, *J* = 7.3, 5.1, 1.0 Hz, 1H, 24-H), 2.62 (*dd*, *J* = 18.6, 3.8 Hz, 1H, 15-H), 2.29 (*td*, *J* = 14.6, 3.6 Hz, 1H, 3-H), 2.14 − 2.07 (*m*, 1H, 6-H), 1.94 (*dt*, *J* = 13.9, 3.7 Hz, 1H, 2-H), 1.86 (*dd*, *J* = 12.8, 3.0 Hz, 1H,6-H), 1.81 (*d*, *J* = 18.6 Hz, 1H, 15-H), 1.78 − 1.77 (*m*, 1H, 1-H), 1.74 (*dt*, *J* = 13.4, 3.3 Hz, 1H, 7-H), 1.71 − 1.67 (*m*, 1H, 11-H), 1.64 − 1.60 (*m*, 1H, 12-H), 1.60 − 1.55 (*m*, 2H, 2-H, 14-H), 1.51 (*dd*, *J* = 13.5, 3.7 Hz, 1H, 7-H), 1.42 (*dd*, *J* = 11.6, 3.8 Hz, 1H, 14-H), 1.40 − 1.35 (*m*, 1H, 12-H), 1.33 (*s*, 3H, 20-H), 1.29 − 1.19 (*m*, 4H, 3-H, 5-H, 9-H, 11-H), 1.02 − 0.98 (*m*, 1H, 1-H), 0.97 (*s*, 3H, 17-H), 0.79 (*s*, 3H, 19-H) ppm; ^13^C NMR (126 MHz, CDCl_3_): δ = 222.23 (C-16), 175.98 (C-18), 151.26 (C-21), 146.07 (C-25), 139.83 (C-23), 119.73 (C-24), 114.85 (C-22), 57.83 (C-5), 54.96 (C-9), 54.33 (C-14), 48.82 (C-13), 48.48 (C-15), 45.20 (C-4), 41.74 (C-7), 40.16 (C-1), 39.62 (C-8), 38.34 (C-3), 38.25 (C-10), 37.39 (C-12), 29.73 (C-20), 22.33 (C-6), 20.49 (C-11), 19.96 (C-17), 19.28 (C-2), 13.78 (C-19) ppm; MS (ESI, MeOH/CHCl_3_, 4:1): *m/z* (%) = 395 (100%, [M+H]^+^), 811 (70%, [2M+Na]^+^); analysis calcd for C_25_H_34_N_2_O_2_ (394.55): C 76.10, H 8.69, N 7.10; found: C 75.96, H 8.83, 6.95.

### 3.6. 16-Oxo-N-pyridin-3-yl-stachan-18-amide (3)

Following GPA from **1** (400 mg, 1.3 mmol), oxalyl chloride (0.55 mL, 6.5 mmol), 3-aminopyridine (612 mg, 6.5 mmol), NEt_3_ (0.72 mL, 5.2 mmol), and chromatography (SiO_2_, CHCl_3_/MeOH, 9:1), **3** (297 mg, 58%) was obtained as a colorless solid; R_f_ = 0.68 (SiO_2_, CHCl_3_/MeOH, 9:1); m.p. 162 °C; ${\left[\alpha \right]}_{D}^{20}$ = −45.77° (*c* = 0.043, MeOH); UV-Vis (MeOH): λ_max_ (log ε) = 240.10 nm (0.52); IR (ATR): ν = 3326*br*, 2926*m*, 2848*m*, 1733*m*, 1664*m*, 1523*m*, 1480*m*, 1414*m*, 1326*w*, 1267*w*, 1191*w*, 1150*w*, 1131*w*, 1109*w*, 1028*w*, 977*w*, 802*w*, 750*s*, 706*m*, 665*w*, 532*w* cm^−1^; ^1^H NMR (500 MHz, CDCl_3_): δ = 8.79 (*s*, 1H, N-H), 8.47 − 8.26 (*m*, 2H, 22-H, 25-H), 7.83 (*s*, 1H, 24-H), 7.38 (*s*, 1H, 23-H), 2.62 (*dd*, *J* = 18.6, 3.7 Hz, 1H, 15-H), 2.28 (*d*, *J* = 14.5 Hz, 1H, 3-H), 2.08 (*d*, *J* = 13.4 Hz, 1H, 6-H), 1.96 − 1.65 (*m*, 6H, 1-H, 2-H, 6-H, 7-H, 11-H, 15-H), 1.65 − 1.48 (*m*, 4H, 2-H, 7-H, 12-H, 14-H), 1.42 (*dd*, *J* = 11.6, 3.7 Hz, 1H, 14-H), 1.40 − 1.36 (*m*, 1H, 12-H), 1.34 (*s*, 3H, 20-H), 1.31 − 1.19 (*m*, 4H, 3-H, 5-H, 9-H, 11-H), 1.01 (*dd*, *J* = 13.2, 4.3 Hz, 1H, 1-H), 0.97 (*s*, 3H, 17-H), 0.80 (*s*, 3H, 19-H) ppm; ^13^C NMR (126 MHz, CDCl_3_): δ = 222.12 (C-16), 176.11 (C-18), 129.68 (C-22), 124.41 (C-23), 57.88 (C-5), 54.96 (C-9), 54.35 (C-14), 48.83 (C-13), 48.49 (C-15), 45.03 (C-4), 41.77 (C-7), 40.16 (C-1), 39.62 (C-8), 38.33 (C-3), 38.28 (C-10), 37.38 (C-12), 29.87 (C-20), 22.41 (C-6), 20.50 (C-11), 19.97 (C-17), 19.37 (C-2), 13.86(C-19) ppm; MS (ESI, MeOH/CHCl_3_, 4:1): *m/z* (%) = 393 (100%, [M-H]^−^), 429 (30%, [M+Cl]^−^); analysis calcd for C_25_H_34_N_2_O_2_ (394.55): C 76.10, H 8.69, N 7.10; found: C 75.97, H 8.91, N 6.96.

### 3.7. 16-Oxo-N-pyridin-4-yl-stachan-18-amide (4)

Following GPA from **1** (400 mg, 1.25 mmol), oxalyl chloride (0.54 mL, 6.3 mmol), 4-aminopyridine (593 mg, 6.3 mmol), NEt_3_ (0.7 mL, 5 mmol), and chromatography (SiO_2_, CHCl_3_/MeOH, 9:1), **4** (335 mg, 68%) was obtained as a colorless solid; R_f_ = 0.27 (SiO_2_, CHCl_3_/MeOH, 9:1); m.p. 155 °C; ${\left[\alpha \right]}_{D}^{20}$ = −59.56° (*c* = 0.160, CHCl_3_); UV-Vis (MeOH): λ_max_ (log ε) = 247.92 nm (0.47); IR (ATR): ν = 2926*m*, 2845*m*, 1732*m*, 1669*m*, 1585*m*, 1504*s*, 1454*m*, 1412*w*, 1325*m*, 1284*w*, 1209*w*, 1127*m*, 1089*w*, 977*w*, 826*m*, 750*s*, 665*w*, *525m* cm^−1^; ^1^H NMR (500 MHz, CDCl_3_): δ = 8.48 (*s*, 1H, N-H), 7.99 (*s*, 2H, 22-H, 25-H), 7.77 (*s*, 2H, 24-H, 23-H), 2.61 (*dd*, *J* = 18.6, 3.7 Hz, 1H, 15-H), 2.30 (*d*, *J* = 14.4 Hz, 1H, 3-H), 2.07 (*t*, *J* = 12.8 Hz, 1H, 6-H), 1.89 − 1.77 (*m*, 5H, 1-H, 2-H, 6-H, 7-H, 11-H), 1.72 (*m*, 3H, 2-H, 7-H, 15-H), 1.64 − 1.48 (*m*, 3H, 12-H, 14-H), 1.46 − 1.36 (*m*, 1H, 12-H), 1.34 (*s*, 3H, 20-H), 1.31 − 1.19 (*m*, 5H, 1-H, 3-H, 5-H, 9-H, 11-H), 0.98 (*s*, 3H, 17-H), 0.77 (*s*, 3H, 19-H) ppm; ^13^C NMR (126 MHz, CDCl_3_): δ = 221.82 (C-16), 176.29 (C-18), 147.93 (C-21), 147.39 (C-23, C-24), 114.30 (C-22, C-25), 57.70 (C-5), 54.81 (C-9), 54.15 (C-14), 48.67 (C-13), 48.30 (C-15), 45.34 (C-4), 41.58 (C-7), 39.92 (C-1), 39.46 (C-8), 38.17 (C-3), 38.12 (C-10), 37.19 (C-12), 29.46 (C-20), 22.21 (C-6), 20.35 (C-11), 19.80 (C-17), 19.12 (C-2), 13.78 (C-19) ppm; MS (ESI, MeOH/CHCl_3_, 4:1): *m/z* (%) = 395 (100%, [M+H]^+^); analysis calcd for C_25_H_34_N_2_O_2_ (394.55): C 76.10, H 8.69, N 7.10; found: C 75.97, H 8.88, N 6.92.

### 3.8. N-(1-Methylpyridinium-2-yl)-16-oxostachan-18-amide Iodide (5)

Following GPB from **2** (223 mg, 0.56 mmol), iodomethane (3.0 mL, 0.05 mmol), and chromatography (SiO_2_, CHCl_3_/MeOH, 9:1), **5** (258 mg, 86%) was obtained as a yellowish solid; R_f_ = 0.82 (SiO_2_, ethyl acetate/MeOH, 8:2); m.p. 155 °C; ${\left[\alpha \right]}_{D}^{20}$ = −114.62° (*c* = 0.032, CHCl_3_); UV-Vis (MeOH): λ_max_ (log ε) = 283.98 nm (0.37); IR (ATR): ν = 2930*m*, 2878*m*, 2832*w*, 1733*s*, 1636*s*, 1596*m*, 1549*m*, 1506*s*, 1444*m*, 1376*s*, 1364*m*, 1320*w*, 1258*w*, 1231*m*, 1177*m*, 1155*s*, 1131*w*, 1105*w*, 1051*w*, 974*w*, 888*w*, 856*m*, 783*m*, 773*m*, 756*m*, 730*w*, 636*w*, 565*w*, 533*w*, 507*w*, 420*w* cm^−1^; ^1^H NMR (500 MHz, CDCl_3_): δ = 7.84 (*d*, *J* = 9.2 Hz, 1H, N-H), 7.43 (*dd*, *J* = 6.9, 1.7 Hz, 1H, 25-H), 7.37 (*m*, 2H, 22-H, 23-H), 6.35 (*td*, *J* = 6.7, 1.4 Hz, 1H, 24-H), 3.67 (*s*, 3H, 26-H), 2.65 (*dd*, *J* = 18.7, 3.8 Hz, 1H, 15-H), 2.37 (*d*, *J* = 12.9 Hz, 1H, 3-H), 2.03 (*d*, *J* = 10.3 Hz, 1H, 6-H), 1.93 (*tdd*, *J* = 14.2, 11.7, 3.2 Hz, 1H, 12-H), 1.77 (*d*, *J* = 18.7 Hz, 1H, 14-H), 1.73 − 1.47 (*m*, 5H, 1-H, 2-H, 6-H, 7-H, 15-H), 1.48 − 1.32 (*m*, 5H, 2-H, 7-H, 11-H, 12-H, 14-H), 1.24 (*s*, 3H, 20-H), 1.23 − 1.12 (*m*, 4H, 3-H, 5-H, 9-H, 11-H), 0.97 (*s*, 3H, 17-H), 0.87 (*d*, *J* = 7.1 Hz, 1H, 1-H), 0.81 (*s*, 3H, 19-H) ppm; ^13^C NMR (126 MHz, CDCl_3_): δ = 223.06 (C-16), 187.23 (C-18), 158.17 (C-21), 138.55 (C-23), 138.47 (C-25), 120.20 (C-24), 109.36 (C-22), 58.06 (C-5), 54.78 (C-9), 54.45 (C-14), 48.76 (C-13), 48.59 (C-15), 46.31 (C-26), 42.14 (C-4), 41.15 (C-7), 40.54 (C-1), 39.60 (C-8), 39.07 (C-3), 38.29 (C-10), 37.49 (C-12), 30.49 (C-20), 22.49 (C-6), 20.41 (C-11), 19.90 (C-17), 19.59 (C-2), 14.11 (C-19) ppm; MS (ESI, MeOH/CHCl_3_ 4:1): *m/z* (%) = 410 (17%, [M+H-I]^+^); analysis calcd for C_26_H_37_N_2_O_2_I (536.50): C 58.21, H 6.95, N 5.22; found: C 57.97, H 7.13, N 5.01.

### 3.9. N-(1-Methylpyridinium-3-yl)-16-oxostachan-18-amide Iodide (6)

Following GPB from **3** (233 mg, 0.59 mmol), iodomethane (3.0 mL, 0.05 mmol), and chromatography (SiO_2_, CHCl_3_/MeOH, 9:1), **6** (294 mg, 93%) was obtained as a yellowish solid; R_f_ = 0.82 (SiO_2_, ethyl acetate/MeOH, 8:2); m.p. 176 °C; ${\left[\alpha \right]}_{D}^{20}$ = 50.44° (*c* = 0.154, CHCl_3_); UV-Vis (MeOH): λ_max_ (log ε) = 220.38 nm (1.10); IR (ATR): ν = 3328*br*, 2926*m*, 2849*m*, 1731*m*, 1684*m*, 1526*m*, 1507*s*, 1451*m*, 1319*m*, 1241*w*, 1153*w*, 1127*w*, 1106*w*, 1034*w*, 963*w*, 748*s*, 699*s*, 592*w*, 529*m* cm^−1^; ^1^H NMR (400 MHz, CDCl_3_): δ = 10.07 (*s*, 1H, 25-H), 9.32 (*d*, *J* = 8.8 Hz, 1H, 22-H), 9.17 (*s*, 1H, N-H), 8.63 (*d*, *J* = 5.8 Hz, 1H, 24-H), 7.90 (*dd*, *J* = 8.7, 5.9 Hz, 1H, 23-H), 4.47 (*s*, 3H, 26-H), 2.66 (*dt*, *J* = 13.8, 2.6 Hz, 1H, 3-H), 2.58 (*dd*, *J* = 18.6, 3.6 Hz, 1H, 15-H), 2.25 − 2.12 (*m*, 1H, 6-H), 1.89 − 1.43 (*m*, 11H, 1-H, 2-H, 6-H, 7-H, 11-H, 12-H, 14-H, 15-H), 1.41 (*s*, 3H, 20-H), 1.39 − 1.11 (*m*, 5H, 3-H, 5-H, 9-H, 11-H, 12-H), 1.02 − 0.97 (*m*, 1H, 1-H), 0.96 (*s*, 3H, 17-H), 0.73 (*s*, 3H, 19-H) ppm; ^13^C NMR (101 MHz, CDCl_3_): δ = 222.57 (C-16), 177.86 (C-18), 140.26 (C-21), 138.56 (C-24), 136.93 (C-25), 136.37 (C-22), 127.80 (C-23), 58.11 (C-5), 54.89 (C-9), 54.28 (C-14), 49.30 (C-26), 48.83 (C-13), 48.62 (C-15), 45.76 (C-4), 41.60 (C-7), 39.91 (C-1), 39.62 (C-8), 38.36 (C-3), 38.07 (C-10), 37.37 (C-12), 29.72 (C-20), 22.61 (C-6), 20.49 (C-11), 19.94 (C-17), 19.68 (C-2), 14.29 (C-19) ppm; MS (ESI, MeOH/CHCl_3_ 4:1): *m/z* (%) = 410 (16%, [M+H-I]^+^); analysis calcd for C_26_H_37_N_2_O_2_I (536.50): C 58.21, H 6.95, N 5.22; found: C 58.01, H 7.17, N 4.96.

### 3.10. N-(1-Methylpyridinium-4-yl)-16-oxostachan-18-amide Iodide (7)

Following GPB from **4** (153 mg, 0.39 mmol), iodomethane (3.0 mL, 0.05 mmol), and chromatography (SiO_2_, CHCl_3_/MeOH, 9:1), **7** (190 mg, 91%) was obtained as a yellowish solid; R_f_ = 0.82 (SiO_2_, ethyl acetate/MeOH, 8:2); m.p. 197 °C; ${\left[\alpha \right]}_{D}^{20}$ = −58.15° (*c* = 0.149, CHCl_3_); UV-Vis (MeOH): λ_max_ (log ε) = 275.94 nm (0.76); IR (ATR): ν = 2926*m*, 2849*m*, 1730*m*, 1671*m*, 1641*m*, 1586*m*, 1515*s*, 1446*m*, 1317*m*, 1198*m*, 1174*w*, 1120*m*, 1086*m*, 1023*w*, 977*w*, 845*w*, 748*s*, 661*w*, 519*m* cm^−1^; ^1^H NMR (400 MHz, CDCl_3_): δ = 9.48 (*s*, 1H, N-H), 8.90 − 8.83 (*m*, 2H, 23-H, 24-H), 8.70 − 8.63 (*m*, 2H, 22-H, 25-H), 4.42 (*s*, 3H, 26-H), 2.75 (*d*, *J* = 14.6 Hz, 1H, 15-H), 2.57 (*dd*, *J* = 18.6, 3.6 Hz, 2H, 1-H, 3-H), 2.22 (*m*, 2H, 6-H, 15-H), 1.86 − 1.63 (*m*, 8H, 2-H, 6-H, 7-H, 11-H, 12-H, 14-H), 1.63 − 1.48 (*m*, 3H, 7-H, 11-H, 14-H), 1.46 (*s*, 3H, 20-H), 1.44 − 1.30 (*m*, 2H, 5-H, 9-H), 1.27 − 1.12 (*m*, 2H, 1-H, 3-H), 0.96 (*s*, 3H, 17-H), 0.70 (*s*, 3H, 19-H) ppm; ^13^C NMR (101 MHz, CDCl_3_): δ = 222.33 (C-16), 178.30 (C-18), 152.95 (C-21), 144.44 (C-23, C-24), 116.98 (C-22, C-25), 58.10 (C-5), 54.84 (C-9), 54.13 (C-14), 48.67 (C-13), 48.44 (C-15), 47.49 (C-26), 46.45 (C-4), 41.46 (C-7), 39.69 (C-1), 39.46 (C-8), 38.21 (C-10), 38.11 (C-3), 37.22 (C-12), 29.16 (C-20), 22.37 (C-6), 20.33 (C-11), 19.80 (C-17), 19.43 (C-2), 14.23 (C-19) ppm; MS (ESI, MeOH/CHCl_3_, 4:1): *m/z* (%) = 410 (36%, [M+H-I]^−^); analysis calcd for C_26_H_37_N_2_O_2_I (536.50): C 58.21, H 6.95, N 5.22; found: C 58.00, H 7.19, N 4.86.

### 3.11. 16-Oxo-N-isoquinolin-4-yl-stachan-18-amide (8)

Following GPA (microwave-assisted) from **1** (250 mg, 0.78 mmol), oxalyl chloride (0.3 mL, 3.12 mmol), 4-amino-isoquinoline (337 mg, 2.34 mmol), NEt_3_ (0.3 mL, 2.34 mmol), and chromatography (SiO_2_, CHCl_3_/MeOH, 9:1), **8** (250 mg, 72%) was obtained as a colorless solid; R_f_ = 0.17 (SiO_2_, hexanes/ethyl acetate, 1:1); m.p. 91 °C; ${\left[\alpha \right]}_{D}^{20}$ = −39.67° (*c* = 0.031, MeOH); UV-Vis (MeOH): λ_max_ (log ε) = 217.81 nm (2.45); IR (ATR): ν = 3320*w*, 2924*m*, 2847*m*, 1734*s*, 1653*m*, 1586*w*, 1511*m*, 1488*s*, 1451*s*, 1410*m*, 1391*m*, 1321*w*, 1278*w*, 1254*w*, 1225*w*, 1169*w*, 1110*w*, 1017*w*, 976*w*, 883*w*, 861*w*, 778*m*, 749*s*, 632*w*, 579*w*, 507*w*, 491*w*, 464*w* cm^−1^; ^1^H NMR (500 MHz, CDCl_3_): δ = 9.13 (*s*, 1H, 23-H), 8.88 (*s*, 1H, 22-H), 8.09 (*d*, *J* = 8.2 Hz, 1H, 25-H), 7.97 (*s*, 1H, N-H), 7.88 − 7.80 (*m*, 2H, 27-H, 28-H), 7.71 (*ddd*, *J* = 8.1, 5.9, 2.0 Hz, 1H, 26-H), 2.63 (*dd*, *J* = 18.6, 3.7 Hz, 1H, 15-H), 2.38 (*dt*, *J* = 15.3, 3.7 Hz, 1H, 3-H), 2.14 − 2.09 (*m*, 1H, 6-H), 2.02 (*dt*, *J* = 13.9, 3.1 Hz, 1H, 2-H), 1.93 (*dd*, *J* = 12.4, 2.9 Hz, 1H, 6-H), 1.88 − 1.83 (*m*, 1H, 1-H), 1.80 (*d*, *J* = 18.6 Hz, 1H, 15-H), 1.76 − 1.69 (*m*, 2H, 7-H, 11-H), 1.69 − 1.66 (*m*, 1H, 2-H), 1.66 − 1.62 (*m*, 1H, 12-H), 1.61 − 1.51 (*m*, 2H, 7-H, 14-H), 1.47 (*s*, 3H, 20-H), 1.44 (*dd*, *J* = 11.7, 3.8 Hz, 1H, 14-H), 1.41 − 1.36 (*m*, 2H, 3-H, 12-H), 1.31 (*dd*, *J* = 12.3, 2.1 Hz, 1H, 5-H), 1.29 − 1.21 (*m*, 2H, 9-H, 11-H), 1.05 (*td*, *J* = 13.6, 4.7 Hz, 1H, 1-H), 0.98 (*s*, 3H, 17-H), 0.89 (*s*, 3H, 19-H) ppm; ^13^C NMR (126 MHz, CDCl_3_): δ = 222.10 (C-16), 176.10 (C-18), 147.58 (C-23), 135.05 (C-22), 132.33 (C-27), 131.60 (C-21), 129.86 (C-29), 129.08 (C-25), 128.61 (C-24), 128.47 (C-26), 120.97 (C-28), 57.80 (C-5), 54.94 (C-9), 54.34 (C-14), 48.82 (C-13), 48.44 (C-15), 45.17 (C-4), 41.79 (C-7), 40.21 (C-1), 39.63 (C-8), 38.54 (C-3), 38.38 (C-10), 37.36 (C-12), 30.35 (C-20), 22.49 (C-6), 20.51 (C-11), 19.96 (C-17), 19.50 (C-2), 14.19 (C-19) ppm; MS (ESI, MeOH/CHCl_3_ 4:1): *m/z* (%) = 443 (100%, [M-H]^−^); analysis calcd for C_29_H_36_N_2_O_2_ (444.62): C 78.34, H 8.16, N 6.30; found: C 78.16, H 7.95, N 6.15.

### 3.12. 16-Oxo-N-quinolin-5-yl-stachan-18-amide (9)

Following GPA (microwave-assisted) from **1** (250 mg (0.78 mmol), oxalyl chloride (0.3 mL (3.12 mmol), 5-aminoquinoline (337 mg, 2.34 mmol), NEt_3_ (0.3 mL, 2.34 mmol), and chromatography (SiO_2_, CHCl_3_/MeOH, 9:1), **9** (187 mg, 54%) was obtained as a colorless solid; R_f_ = 0.15 (SiO_2_, hexanes/ethyl acetate, 1:1); m.p. 90 °C; ${\left[\alpha \right]}_{D}^{20}$ = −35.71° (*c* = 0.056, MeOH); UV-Vis (MeOH): λ_max_ (log ε) = 229.71 nm (0.86); IR (ATR): ν = 3337*w*, 2924*m*, 2847*m*, 1734*m*, 1649*m*, 1594*w*, 1510*w*, 1485*m*, 1452*m*, 1397*w*, 1318*w*, 1261*w*, 1171*w*, 1131*w*, 1110*w*, 976*w*, 862*w*, 798*s*, 750*s*, 656*w*, 589*w*, 498*w*, 467*w* cm^−1^; ^1^H NMR (500 MHz, CDCl_3_): δ = 8.78 (*dd*, *J* = 4.5, 1.5 Hz, 1H, 26-H), 8.31 (*d*, *J* = 8.4 Hz, 1H, 22-H), 8.02 (*d*, *J* = 8.1 Hz, 1H, N-H), 7.77 (*dd*, *J* = 7.6, 1.1 Hz, 2H, 27-H, 28-H), 7.71 (*dd*, *J* = 8.4, 7.5 Hz, 1H, 24-H), 7.49 (*dd*, *J* = 8.5, 4.4 Hz, 1H, 23-H), 2.64 (*dd*, *J* = 18.5, 3.7 Hz, 1H, 15-H), 2.37 (*d*, *J* = 14.4 Hz, 1H, 3-H), 2.09 (*td*, *J* = 13.8, 6.9 Hz, 1H, 6-H), 2.05 − 1.84 (*m*, 3H, 1-H, 2-H, 6-H), 1.80 (*d*, *J* = 18.6 Hz, 1H, 15-H), 1.77 − 1.59 (*m*, 4H, 2-H, 7-H, 11-H, 12-H), 1.60 − 1.51 (*m*, 2H, 7-H, 14-H), 1.47 (*s*, 3H, 20-H), 1.44 − 1.33 (*m*, 3H, 3-H, 12-H, 14-H), 1.34 − 1.19 (*m*, 3H, 5-H, 9-H, 11-H), 1.05 (*td*, *J* = 13.2, 4.4 Hz, 1H, 1-H), 0.98 (*s*, 3H, 17-H), 0.91 (*s*, 3H, 19-H) ppm; ^13^C NMR (126 MHz, CDCl_3_): δ = 222.03 (C-16), 176.18 (C-18), 148.08 (C-23), 133.47 (C-22), 130.62 (C-27), 125.31 (C-21), 124.06 (C-29), 123.41 (C-25, C-26), 120.78 (C-24, C-28), 57.72 (C-5), 54.77 (C-9), 54.20 (C-14), 48.69 (C-13), 48.34 (C-15), 44.89 (C-4), 41.66 (C-7), 40.09 (C-1), 39.51 (C-8), 38.38 (C-3), 38.24 (C-10), 37.24 (C-12), 30.28 (C-20), 22.36 (C-6), 20.38 (C-11), 19.82 (C-17), 19.40 (C-2), 14.09 (C-19) ppm; MS (ESI, MeOH/CHCl_3_, 4:1): *m/z* (%) = 445 (90%, [M+H]^+^), 911 (100%, [2M+Na]^+^); analysis calcd for C_29_H_36_N_2_O_2_ (444.62): C 78.34, H 8.16, N 6.30; found: C 78.07, H 8.36, N 6.13.

### 3.13. N-(1-Methylisoquinolinium-4-yl)-16-oxostachan-18-amide Iodide (10)

Following GPB from **8** (97 mg, 0.22 mmol), iodomethane (3.0 mL, 0.05 mmol), and chromatography (SiO_2_, CHCl_3_/MeOH, 9:1), **10** (93 mg, 72%) was obtained a yellowish solid; R_f_ = 0.15 (SiO_2_, hexanes/ethyl acetate, 1:1); m.p. 188 °C; ${\left[\alpha \right]}_{D}^{20}$ = −46.89° (*c* = 0.110, CHCl_3_); UV-Vis (MeOH): λ_max_ (log ε) = 228.82 nm (1.50); IR (ATR): ν = 3225*w*, 3078*w*, 2926*m*, 2841*m*, 1730*s*, 1690*m*, 1643*w*, 1608*w*, 1519*m*, 1499*m*, 1472*m*, 1445*s*, 1415*w*, 1352*m*, 1259*m*, 1219*w*, 1187*w*, 1157*m*, 1133*w*, 1109*w*, 980*w*, 868*w*, 783*m*, 752*w*, 638*w*, 590*m*, 553*w*, 524*w*, 512*w*, 445*w*, 425*w* cm^−1^; ^1^H NMR (400 MHz, CDCl_3_): δ = 10.40 (*s*, 1H, 23-H), 9.06 (*d*, *J* = 1.4 Hz, 1H, 22-H), 8.76 (*s*, 1H, N-H), 8.54 (*d*, *J* = 8.3 Hz, 1H, 28-H), 8.19 − 8.08 (*m*, 2H, 25-H, 26-H), 7.92 (*ddd*, *J* = 8.2, 4.9, 3.1 Hz, 1H, 27-H), 4.52 (*s*, 3H, 30-H), 2.65 − 2.53 (*m*, 2H, 3-H, 15-H), 2.21 − 2.12 (*m*, 1H, 6-H), 1.97 − 1.92 (*m*, 1H, 2-H), 1.89 (*dd*, *J* = 12.8, 2.9 Hz, 1H, 6-H), 1.81 (*d*, *J* = 18.7 Hz, 1H, 15-H), 1.81 − 1.65 (*m*, 4H, 1-H, 2-H, 7-H, 11-H), 1.63 − 1.52 (*m*, 3H, 7-H, 12-H, 14-H), 1.49 (*s*, 3H, 20-H), 1.43 (*dd*, *J* = 11.8, 3.7 Hz, 1H, 14-H), 1.40 − 1.34 (*m*, 2H, 3-H, 12-H), 1.32 (*dd*, *J* = 12.4, 2.0 Hz, 1H, 5-H), 1.28 − 1.22 (*m*, 1H, 9-H), 1.19 (*dd*, *J* = 12.7, 5.2 Hz, 1H, 11-H), 1.04 (*td*, *J* = 13.1, 4.3 Hz, 1H, 1-H), 0.96 (*s*, 3H, 17-H), 0.79 (*s*, 3H, 19-H) ppm; ^13^C NMR (101 MHz, CDCl_3_): δ = 222.21 (C-16), 177.11 (C-18), 145.95 (C-23), 136.91 (C-26), 134.29 (C-29), 131.83 (C-21), 131.53 (C-27), 131.31 (C-28), 129.06 (C-22), 127.99 (C-24), 122.37 (C-25), 57.92 (C-5), 54.83 (C-9), 54.24 (C-14), 48.89 (C-30), 48.82 (C-13), 48.49 (C-15), 45.73 (C-4), 41.66 (C-7), 39.97 (C-1), 39.63 (C-8), 38.37 (C-3), 38.32 (C-10), 37.32 (C-12), 30.19 (C-20), 22.54 (C-6), 20.49 (C-11), 19.93 (C-17), 19.64 (C-2), 14.48 (C-19) ppm; MS (ESI, MeOH/CHCl_3_, 4:1): *m/z* (%) = 460 (21%, [M+H-I]^+^); analysis calcd for C_30_H_39_N_2_O_2_I (586.56): C 61.43, H 6.70, N 4.78; found: C 71.20, H 6.97, N 4.44.

### 3.14. N-(1-Methylquinolinium-5-yl)-16-oxostachan-18-amide Iodide (11)

Following GPB from **9** (60 mg, 0.14 mmol), iodomethane (3.0 mL, 0.05 mmol), and chromatography (SiO_2_, CHCl_3_/MeOH, 9:1), **11** (65 mg, 79%) was obtained as a yellowish solid; R_f_ = 0.53 (SiO_2_, CHCl_3_/MeOH, 8:2); m.p. 195 °C; ${\left[\alpha \right]}_{D}^{20}$ = −61.93° (*c* = 0.108, MeOH); UV-Vis (MeOH): λ_max_ (log ε) = 219.17 nm (0.92); IR (ATR): ν = 3428*m*, 2924*m*, 2848*m*, 1732*s*, 1667*w*, 1622*m*, 1592*m*, 1533*m*, 1492*m*, 1450*s*, 1406*w*, 1368*w*, 1336*w*, 1279*w*, 1244*m*, 1161*m*, 1128*w*, 1111*w*, 1088*w*, 1029*w*, 977*w*, 929*w*, 862*w*, 793*s*, 750*m*, 696*w*, 559*w*, 527*m*, 463*m* cm^−1^; ^1^H NMR (500 MHz, CDCl_3_): δ = 9.76 (*d*, *J* = 5.7 Hz, 1H, 28-H), 9.28 (*d*, *J* = 8.6 Hz, 1H, 26-H), 8.77 (*s*, 1H, N-H), 8.22 − 8.05 (*m*, 2H, 22-H, 27-H), 7.98 (*dd*, *J* = 8.6, 5.7 Hz, 2H, 23-H, 24-H), 4.71 (*s*, 3H, 30-H), 2.72 − 2.61 (*m*, 1H, 15-H), 2.59 (*d*, *J* = 3.6 Hz, 1H, 3-H), 2.16 (*d*, *J* = 13.4 Hz, 1H, 6-H), 2.00 − 1.78 (*m*, 4H, 1-H, 2-H, 6-H, 15-H), 1.77 − 1.55 (*m*, 6H, 2-H, 7-H, 11-H, 12-H, 14-H), 1.52 (*s*, 3H, 20-H), 1.49 − 1.30 (*m*, 3H, 3-H, 12-H, 14-H), 1.30 − 1.18 (*m*, 3H, 5-H, 9-H, 11-H), 1.05 (*td*, *J* = 13.4, 4.4 Hz, 1H, 1-H), 0.98 (*s*, 3H, 17-H), 0.87 (*s*, 3H, 19-H) ppm; ^13^C NMR (126 MHz, CDCl_3_): δ = 222.33 (C-16), 177.27 (C-18), 149.55 (C-23), 144.31 (C-22), 139.08 (C-27), 136.96 (C-21), 136.19 (C-29), 127.35 (C-26), 126.15 (C-25), 120.98 (C-24), 115.24 (C-28), 57.94 (C-5), 54.77 (C-9), 54.19 (C-14), 48.71 (C-13, C-15), 48.43 (C-30), 46.84 (C-4), 45.23 (C-7), 41.61 (C-1), 39.97 (C-8), 39.53 (C-3), 38.27 (C-10), 37.27 (C-12), 30.20 (C-20), 22.46 (C-6), 20.39 (C-11), 19.83 (C-17), 19.80 (C-2), 14.38 (C-19) ppm; MS (ESI, MeOH/CHCl_3_, 4:1): *m/z* (%) = 460 (35%, [M+H-I]^+^); analysis calcd for C_30_H_39_N_2_O_2_I (586.56): C 61.43, H 6.70, N 4.78; found: C 61.26, H 6.91, N 4.55.

### 3.15. 16-Oxo-N-phenyl-stachan-18-amide (12)

Following GPA from **1** (300 mg, 0.94 mmol), aniline (0.35 mL, 3.76 mmol), and chromatography (SiO_2_, CHCl_3_/MeOH, 9:1), **12** (237 mg, 64%) was obtained as an off-white solid; m.p. = 65 °C; ${\left[\alpha \right]}_{D}^{20}$ = −57.68° (*c* = 0.108, MeOH); UV-Vis (MeOH): λ_max_ (log ε) = 238.27 nm (1.23); IR (ATR): ν = 3371*w*, 2925*m*, 2847*m*, 1730*s*, 1667*m*, 1595*w*, 1519*w*, 1499*s*, 1434*s*, 1306*m*, 1237*w*, 1150*w*, 1131*w*, 1109*w*, 1028*w*, 976*w*, 748*s*, 691*s*, 596*w*, 527*m* cm^−1^; ^1^H NMR (500 MHz, CDCl_3_): δ = 7.47 − 7.43 (m, 2H, 22-H, 26-H), 7.33 − 7.27 (m, 2H, 23-H, 25-H), 7.12 − 7.07 (m, 1H, 24-H), 6.68 − 6.71 (m, 1H, N-H), 2.63 (dd, *J* = 18.6, 3.8 Hz, 1H, 15-H), 2.18 (dd, *J* = 14.6, 2.1 Hz, 1H, 3-H), 2.08 − 2.01 (m, 1H, 6-H), 1.95 − 1.89 (m, 1H, 2-H), 1.86 (dd, *J* = 12.7, 3.1 Hz, 1H, 6-H), 1.81 (s, 1H, 1-H), 1.80 (d, *J* = 18.7 Hz, 1H, 15-H), 1.72 (dt, *J* = 13.6, 3.4 Hz, 1H, 7-H), 1.70 − 1.67 (m, 1H, 11-H), 1.63 − 1.59 (m, 1H, 12-H), 1.57 − 1.47 (m, 3H, 2-H, 7-H, 14-H), 1.41 − 1.37 (m, 1H, 12-H), 1.30 (s, 3H, 20-H), 1.27 − 1.19 (m, 4H, 3-H, 5-H, 9-H, 11-H), 1.03 − 0.99 (m, 1H, 1-H), 0.98 (s, 3H, 17-H), 0.82 (s, 3H,19-H) ppm; ^13^C NMR (126 MHz, CDCl_3_): δ = 222.07 (C-16), 174.88 (C-18), 137.82 (C-21), 129.00 (C-23, C-25), 124.35 (C-24), 120.41 (C-22, C-26), 57.69 (C-5), 54.80 (C-9), 54.21 (C-14), 48.69 (C-13), 48.32 (C-15), 44.53 (C-4), 41.69 (C-7), 40.14 (C-1), 39.49 (C-8), 38.27 (C-3), 38.17 (C-10), 37.25 (C-12), 29.91 (C-20), 22.30 (C-6), 20.36 (C-11), 19.84 (C-17), 19.17 (C-2), 13.62 (C-19) ppm; MS (ESI, MeOH/CHCl_3_, 4:1): *m/z* (%) = 392 (100%, [M-H]^−^); analysis calcd for C_26_H_35_NO_2_ (393.57): C 79.35, H 8.96, N 3.56; found: C 79.17, H 9.18, N 3.23.

### 3.16. N-Benzyl-16-oxostachan-18-amide (13)

Following GPA from **1** (250 mg, 0.78 mmol), benzylamine (0.44 mL, 4.0 mmol), and chromatography (SiO_2_, hexanes/ethyl acetate, 95:5), **13** (210 mg, 66%) was obtained as a colorless solid; R_f_ = 0.28 (SiO_2_, hexanes/ethyl acetate, 8:2); m.p. = 71 °C; ${\left[\alpha \right]}_{D}^{20}$ = −65.44° (*c* = 0.122, MeOH); IR (ATR): ν = 3371*w*, 2925*m*, 2847*m*, 1730*s*, 1667*m*, 1595*w*, 1519*w*, 1499*s*, 1434*s*, 1306*m*, 1237*w*, 1150*w*, 1131*w*, 1109*w*, 1028*w*, 976*w*, 748*s*, 691*s*, 596*w*, 527*m* cm^−1^; ^1^H NMR (500 MHz, CDCl_3_): δ = 7.36 − 7.26 (m, 5H, 23-H, 24-H, 25-H, 26-H, 27-H), 5.86 (t, *J* = 5.5 Hz, 1H, N-H), 4.40 (dd, *J* = 5.5, 1.7 Hz, 2H, 21-H), 2.63 (dd, *J* = 18.7, 3.8 Hz, 1H, 15-H), 2.06 − 2.00 (m, 1H, 3-H), 1.97 − 1.92 (m, 1H, 6-H), 1.84 − 1.71 (m, 4H, 1-H, 2-H, 6-H, 15-H), 1.69 − 1.63 (m, 2H, 7-H, 11-H), 1.62 − 1.57 (m, 1H, 12-H), 1.54 (dd, *J* = 11.6, 2.7 Hz, 1H, 14-H), 1.50 − 1.42 (m, 2H, 2-H, 7-H), 1.41 − 1.32 (m, 2H, 12-H, 14-H), 1.21 (s, 3H, 20-H), 1.20 − 1.10 (m, 4H, 3-H, 5-H, 9-H, 11-H), 0.97 (s, 3H, 17-H), 0.96 − 0.89 (m, 1H, 1-H), 0.74 (s, 3H, 19-H) ppm; ^13^C NMR (126 MHz, CDCl_3_): δ = 222.50 (C-16), 176.51 (C-18), 138.64 (C-22), 128.85 (C-24, C-26), 128.11 (C-23, C-27), 127.63 (C-25), 57.77 (C-5), 54.89 (C-9), 54.41 (C-14), 48.84 (C-13), 48.52 (C-15), 43.85 (C-4, C-21), 41.84 (C-7), 40.30 (C-1), 39.63 (C-8), 38.27 (C-3), 38.23 (C-10), 37.43 (C-12), 30.34 (C-20), 22.40 (C-6), 20.47 (C-11), 19.99 (C-17), 19.36 (C-2), 13.74 (C-19) ppm; MS (ESI, MeOH/CHCl_3_ 4:1): *m/z* (%) = 408 (100%, [M+H]^+^), 430 (95%, [M+Na]^+^), 837 (80%, [2M+Na]^+^); analysis calcd for C_27_H_37_NO_2_ (407.60): C 79.56, H 9.15, N 3.44; found: C 79.31, H 9.36, N 3.20.

### 3.17. 16-Oxo-N-(2-phenylethyl)-stachan-18-amide (14)

Following GPA from **1** (500 mg, 1.57 mmol), phenethylamine (0.8 mL, 6.28 mmol), and chromatography (SiO_2_, hexanes/ethyl acetate, 95:5), **14** (470 mg, 71%) was obtained as a colorless solid; R_f_ = 0.18 (SiO_2_, hexanes/ethyl acetate, 8:2); m.p. = 82 °C; ${\left[\alpha \right]}_{D}^{20}$ = −53.68° (*c* = 0.106, MeOH); UV-Vis (MeOH): λ_max_ (log ε) = 213.81 nm (3.41); IR (ATR): ν = 3371*w*, 2925*m*, 2847*m*, 1730*s*, 1667*m*, 1595*w*, 1519*w*, 1499*s*, 1434*s*, 1306*m*, 1237*w*, 1150*w*, 1131*w*, 1109*w*, 1028*w*, 976*w*, 748*s*, 691*s*, 596*w*, 527*m* cm^−1^; ^1^H NMR (500 MHz, CDCl_3_): δ = 7.34 − 7.07 (m, 5H, 24-H, 25-H, 26-H, 27-H, 28-H), 5.56 (s, 1H, N-H), 3.57 − 3.43 (m, 2H, 21-H), 2.88 − 2.72 (m, 2H, 22-H), 2.55 (dd, *J* = 18.7, 3.7 Hz, 1H, 15-H), 1.94 (d, *J* = 14.1 Hz, 1H, 3-H), 1.82 − 1.76 (m, 1H, 6-H), 1.72 (d, *J* = 18.7 Hz, 1H, 15-H), 1.69 − 1.60 (m, 3H, 1-H, 2-H, 11-H), 1.59 − 1.53 (m, 3H, 6-H, 7-H, 12-H), 1.50 (dd, *J* = 11.6, 2.7 Hz, 2H, 14-H), 1.41 (dd, *J* = 13.2, 3.3 Hz, 1H, 7-H), 1.38 − 1.29 (m, 3H, 2-H, 12-H, 14-H), 1.17 (dd, *J* = 12.9, 5.2 Hz, 1H, 11-H), 1.14 − 1.12 (m, 1H, 9-H), 1.10 (s, 3H, 20-H), 1.06 − 1.02 (m, 2H, 3-H, 5-H), 0.95 (s, 3H, 17-H), 0.85 (td, *J* = 13.4, 12.0, 6.9 Hz, 1H, 1-H), 0.66 (s, 3H, 19-H) ppm; ^13^C NMR (126 MHz, CDCl_3_): δ = 222.36 (C-16), 176.76 (C-18), 139.03 (C-23), 128.80 (C-24, C-25, C-27, C-28), 126.65 (C-26), 57.44 (C-5), 54.78 (C-9), 54.30 (C-14), 48.73 (C-13), 48.54 (C-15), 43.70 (C-4), 41.73 (C-7), 40.52 (C-21), 40.23 (C-1), 39.51 (C-8), 38.17 (C-3), 38.11 (C-10), 37.34 (C-12), 35.25 (C-22), 30.24 (C-20), 22.14 (C-6), 20.37 (C-11), 19.92 (C-17), 19.12 (C-2), 13.52 (C-19) ppm; MS (ESI, MeOH/CHCl_3_, 4:1): *m/z* (%) = 420 (100%, [M-H]^−^); analysis calcd for C_28_H_39_NO_2_ (421.63): C 79.63, H 9.32, N 3.32; found: C 79.41, H 9.57, N 3.01.

### 3.18. N-(2-Fluorobenzyl)-16-oxostachan-18-amide (15)

Following GPA from **1** (300 mg, 0.94 mmol), 2-fluorobenzylamine (0.32 mL, 2.82 mmol), and chromatography (SiO_2_, hexanes/ethyl acetate, 95:5), **15** (368 mg, 92%) was obtained as a colorless solid; R_f_ = 0.32 (SiO_2_, hexanes/ethyl acetate, 8:2); m.p. = 60 °C; ${\left[\alpha \right]}_{D}^{20}$ = −51.6° (*c* = 0.05, MeOH); UV-Vis (MeOH): λ_max_ (log ε) = 238.27 nm (1.23); IR (ATR): ν = 3371*w*, 2925*m*, 2847*m*, 1730*s*, 1667*m*, 1595*w*, 1519*w*, 1499*s*, 1434*s*, 1306*m*, 1237*w*, 1150*w*, 1131*w*, 1109*w*, 1028*w*, 976*w*, 748*s*, 691*s*, 596*w*, 527*m* cm^−1^; ^1^H NMR (500 MHz, CDCl_3_): δ = 7.38 − 7.33 (m, 1H, 23-H), 7.28 − 7.22 (m, 1H, 25-H), 7.10 − 7.06 (m, 1H, 24-H), 7.06 − 7.01 (m, 1H, 26-H), 6.00 (t, *J* = 5.7 Hz, 1H, N-H), 4.46 − 4.37 (m, 2H, 21-H), 2.56 (dd, *J* = 18.7, 3.8 Hz, 1H, 15-H), 2.06 − 2.01 (m, 1H, 3-H), 1.95 − 1.90 (m, 1H, 6-H), 1.82 − 1.73 (m, 3H, 2-H, 6-H, 15-H), 1.72 − 1.61 (m, 3H, 1-H, 7-H, 11-H), 1.60 − 1.55 (m, 1H, 12-H), 1.52 (dd, *J* = 11.6, 2.8 Hz, 1H, 14-H), 1.49 − 1.42 (m, 2H, 2-H, 7-H), 1.38 (dd, *J* = 11.6, 3.9 Hz, 1H, 14-H), 1.35 − 1.30 (m, 1H, 12-H), 1.17 (s, 3H, 20-H), 1.16 − 1.12 (m, 3H, 3-H, 9-H, 11-H), 1.10 (dd, *J* = 12.2, 2.2 Hz, 1H, 5-H), 0.96 (s, 3H, 17-H), 0.94 − 0.85 (m, 1H, 1-H), 0.55 (s, 3H, 19-H) ppm; ^13^C NMR (126 MHz, CDCl_3_): ^13^C NMR (126 MHz, CDCl_3_): δ = 222.55 (C-16), 176.57 (C-18), 161.36 (*d*, *J* = 245.4 Hz, C-27), 131.14 (*d*, *J* = 4.4 Hz, C-23), 129.47 (*d*, *J* = 8.2 Hz, C-25), 125.37 (*d*, *J* = 14.7 Hz, C-22), 124.40 (*d*, *J* = 3.8 Hz, C-24), 115.37 (*d*, *J* = 21.2 Hz, C-26), 57.71 (C-5), 54.84 (C-9), 54.39 (C-14), 48.83 (C-13), 48.47 (C-15), 43.85 (C-4), 41.82 (C-7), 40.27 (C-1), 39.61 (C-10), 38.20 (C-3), 38.15 (C-8), 38.10 (C-21), 37.43 (C-12), 30.19 (C-20), 22.27 (C-6), 20.44 (C-11), 19.98 (C-17), 19.22 (C-2), 13.27 (C-19) ppm; ^19^F NMR (470 MHz, CDCl_3_): δ = −119.37 − 119.47 (*m*) ppm; MS (ESI, MeOH/CHCl_3_, 4:1): *m/z* (%) = 424 (100%, [M-H]^−^); analysis calcd for C_27_H_36_FNO_2_ (425.59): C 76.20, H 8.53, N 3.29; found: C 75.96, H 8.77, N 2.97.

### 3.19. N-(3-Fluorobenzyl)-16-oxostachan-18-amide (16)

Following GPA from **1** (300 mg, 0.94 mmol), 3-fluorobenzylamine (0.32 mL, 2.82 mmol), and chromatography (SiO_2_, hexanes/ethyl acetate, 95:5), **16** (304 mg, 38%) was obtained as a colorless solid; R_f_ = 0.32 (SiO_2_, hexanes/ethyl acetate, 8:2); m.p. = 60 °C; ${\left[\alpha \right]}_{D}^{20}$ = −51.6° (*c* = 0.05, MeOH); UV-Vis (MeOH): λ_max_ (log ε) = 238.27 nm (1.23); IR (ATR): ν = 3371*w*, 2925*m*, 2847*m*, 1730*s*, 1667*m*, 1595*w*, 1519*w*, 1499*s*, 1434*s*, 1306*m*, 1237*w*, 1150*w*, 1131*w*, 1109*w*, 1028*w*, 976*w*, 748*s*, 691*s*, 596*w*, 527*m* cm^−1^; ^1^H NMR (500 MHz, CDCl_3_): δ = 7.31 − 7.26 (m, 1H, 24-H), 7.07 − 7.02 (m, 1H, 23-H), 6.99 − 6.92 (m, 2H, 25-H, 27-H), 5.93 (t, *J* = 5.8 Hz, 1H, N-H), 4.44 − 4.34 (m, 2H, 21-H), 2.61 (dd, *J* = 18.6, 3.8 Hz, 1H, 15-H), 2.07 − 2.01 (m, 1H, 3-H), 1.97 − 1.92 (m, 1H, 6-H), 1.83 − 1.72 (m, 4H, 1-H, 2-H, 6-H, 15-H), 1.70 − 1.64 (m, 2H, 7-H, 11-H), 1.62 − 1.57 (m, 1H, 12-H), 1.54 (dd, *J* = 11.6, 2.8 Hz, 1H, 14-H), 1.50 − 1.43 (m, 2H, 2-H, 7-H), 1.40 (dd, *J* = 11.7, 4.0 Hz, 1H, 14-H), 1.35 (dd, *J* = 12.6, 5.2 Hz, 1H, 12-H), 1.21 (s, 3H, 3-H, 9-H, 11-H), 1.27 − 1.16 (m, 3H, 20-H), 1.13 (dd, *J* = 12.3, 2.2 Hz, 1H, 5-H), 0.96 (s, 3H, 17-H), 0.95 − 0.88 (m, 1H, 1-H), 0.72 (s, 3H, 19-H) ppm; ^13^C NMR (126 MHz, CDCl_3_): δ = 222.44 (C-16), 176.65 (C-18), 164.09 (*d*, *J* = 246.4 Hz, C-26), 141.30 (*d*, *J* = 7.1 Hz, C-22), 130.33 (*d*, *J* = 8.1 Hz, C-24), 123.53 (*d*, *J* = 2.9 Hz, C-23), 114.86 (*d*, *J* = 21.5 Hz, C-27), 114.47 (*d*, *J* = 21.0 Hz, C-25), 57.73 (C-5), 54.88 (C-9), 54.39 (C-14), 48.83 (C-13), 48.50 (C-15), 43.91 (C-4), 43.24 (C-21), 41.82 (C-7), 40.26 (C-1), 39.61 (C-8), 38.25 (C-3), 38.22 (C-10), 37.42 (C-12), 30.33 (C-20), 22.40 (C-6), 20.47 (C-11), 19.98 (C-17), 19.35 (C-2), 13.74 (C-19) ppm; ^19^F NMR (470 MHz, CDCl_3_): δ = -112.73 (*td*, *J* = 9.2, 5.9 Hz) ppm; MS (ESI, MeOH/CHCl_3_, 4:1): *m/z* (%) = 424 (100%, [M-H]^−^); analysis calcd for C_27_H_36_FNO_2_ (425.59): C 76.20, H 8.53, N 3.29; found: C 75.86, H 8.71, N 3.11.

### 3.20. N-(4-Fluorobenzyl)-16-oxostachan-18-amide (17)

Following GPA from **1** (250 mg, 0.78 mmol), 4-fluoro-benzylamine (0.27 mL, 2.34 mmol), and chromatography (SiO_2_, hexanes/ethyl acetate, 95:5), **17** (302 mg, 91%) was obtained as a colorless solid; R_f_ = 0.2 (SiO_2_, hexanes/ethyl acetate, 8:2); m.p. = 62 °C; ${\left[\alpha \right]}_{D}^{20}$ = −87.89° (*c* = 0.09, MeOH); UV-Vis (MeOH): λ_max_ (log ε) = 211 nm (3.10); IR (ATR): ν = 3371*w*, 2925*m*, 2847*m*, 1730*s*, 1667*m*, 1595*w*, 1519*w*, 1499*s*, 1434*s*, 1306*m*, 1237*w*, 1150*w*, 1131*w*, 1109*w*, 1028*w*, 976*w*, 748*s*, 691*s*, 596*w*, 527*m* cm^−1^; ^1^H NMR (400 MHz, CDCl_3_): δ = 7.27 − 7.20 (m, 2H, 23-H, 27-H), 7.05 − 6.93 (m, 2H, 24-H, 26-H), 5.88 (t, *J* = 5.7 Hz, 1H, N-H), 4.43 − 4.29 (m, 2H, 21-H), 2.62 (dd, *J* = 18.6, 3.7 Hz, 1H, 15-H), 2.06 − 1.98 (m, 1H, 3-H), 1.98 − 1.90 (m, 1H, 6-H), 1.83 − 1.63 (m, 6H, 1-H, 2-H, 6-H, 7-H, 11-H, 15-H), 1.62 − 1.57 (m, 1H, 12-H), 1.54 (dd, *J* = 11.6, 2.7 Hz, 1H, 14-H), 1.50 − 1.31 (m, 4H, 2-H, 7-H, 12-H, 14-H), 1.28 − 1.21 (m, 2H, 3-H, 11-H), 1.20 (s, 3H, 20-H), 1.18 (s, 1H, 9-H), 1.13 (dd, *J* = 12.1, 2.2 Hz, 1H, 5-H), 0.96 (s, 3H, 17-H), 0.90 (dd, *J* = 13.8, 3.9 Hz, 1H, 1-H), 0.72 (s, 3H, 19-H) ppm; ^13^C NMR (101 MHz, CDCl_3_): δ = 222.45 (C-16), 176.55 (C-18), 162.28 (*d*, *J* = 245.7 Hz, C-25), 134.50 (*d*, *J* = 3.0 Hz, C-22), 129.76 (*d*, *J* = 7.6 Hz, C-23, C-27), 115.66 (*d*, *J* = 21.4 Hz, C-24, C-26), 57.74 (C-5), 54.86 (C-9), 54.39 (C-14), 48.82 (C-13), 48.51 (C-15), 43.86 (C-4), 43.06 (C-21), 41.82 (C-7), 40.27 (C-1), 39.62 (C-8), 38.23 (C-3, C-10), 37.41 (C-12), 30.31 (C-20), 22.38 (C-6), 20.47 (C-11), 19.97 (C-17), 19.34 (C-2), 13.75 (C-19) ppm; ^19^F NMR (470 MHz, CDCl_3_): δ = -114.97 (*ddd*, *J* = 14.0, 8.8, 5.2 Hz) ppm; MS (ESI, MeOH/CHCl_3_, 4:1): *m/z* (%) = 424 (100%, [M-H]^−^); analysis calcd for C_27_H_36_FNO_2_ (425.59): C 76.20, H 8.53, N 3.29; found: C 75.87, H 8.76, N 3.03.

### 3.21. N-(2-Methylbenzyl)-16-oxostachan-18-amide (18)

Following GPA from **1** (465 mg, 1.46 mmol), 2-methyl-benzylamine (0.75 mL, 5.8 mmol), and chromatography (SiO_2_, CHCl_3_/MeOH, 9:1), **18** (425 mg, 69%) was obtained as a colorless solid; R_f_ = 0.87 (SiO_2_, CHCl_3_/MeOH, 9:1); m.p. = 75 °C; ${\left[\alpha \right]}_{D}^{20}$ = −57.17° (*c* = 0.16, CHCl_3_); IR (ATR): ν = 3391*w*, 2924*m*, 2847*m*, 1732*m*, 1644*m*, 1511*m*, 1454*m*, 1238*w*, 1189*w*, 1109*w*, 1005*w*, 976*w*, 740*s*, 695*w*, 665*w*, 589*w*, 506*w*, 456*w*, 430*w* cm^−1^; ^1^H NMR (500 MHz, CDCl_3_): δ = 7.25 − 7.09 (m, 4H, 23-H, 24-H, 25-H, 26-H), 5.67 (t, *J* = 5.2 Hz, 1H, N-H), 4.46 − 4.32 (m, 2H, 21-H), 2.63 (dd, *J* = 18.6, 3.8 Hz, 1H, 15-H), 2.33 (s, 3H, 28-H), 2.07 − 1.98 (m, 1H, 3-H), 1.98 − 1.90 (m, 1H, 6-H), 1.85 − 1.56 (m, 7H, 1-H, 2-H, 6-H, 7-H, 11-H, 12-H, 15-H), 1.54 (dd, *J* = 11.6, 2.8 Hz, 1H, 14-H), 1.50 − 1.32 (m, 4H, 2-H, 7-H, 12-H, 14-H), 1.21 (s, 3H, 20-H), 1.19 (s, 3H, 3-H, 9-H, 11-H), 1.13 (dd, *J* = 12.1, 2.1 Hz, 1H, 5-H), 0.97 (s, 3H, 17-H), 0.95 − 0.89 (m, 1H, 1-H), 0.76 (s, 3H, 19-H) ppm; ^13^C NMR (126 MHz, CDCl_3_): δ = 222.49 (C-16), 176.46 (C-18), 136.58 (C-22), 136.19 (C-27), 130.73 (C-26), 128.97 (C-23), 127.93 (C-25), 126.38 (C-24), 57.81 (C-5), 54.91 (C-9), 54.43 (C-14), 48.85 (C-13), 48.53 (C-15), 43.93 (C-4), 42.01 (C-21), 41.86 (C-7), 40.33 (C-1), 39.64 (C-8), 38.28 (C-3), 38.22 (C-10), 37.45 (C-12), 30.39 (C-20), 22.39 (C-6), 20.49 (C-11), 20.00 (C-17), 19.39 (C-2), 19.20 (C-28), 13.73 (C-19) ppm; MS (ESI, MeOH/CHCl_3_, 4:1): *m/z* (%) = 420 (100%, [M-H]^−^); analysis calcd for C_28_H_39_NO_2_ (421.63): C 79.76, H 9.32, N 3.32; found: C 79.51, H 9.48, N 3.09.

### 3.22. N-(3-Methylbenzyl)-16-oxostachan-18-amide (19)

Following GPA from **1** (418 mg, 1.31 mmol), 3-methyl-benzylamine (0.66 mL, 3.24 mmol) and chromatography (SiO_2_, CHCl_3_/MeOH, 9:1), **19** (453 mg, 82%) was obtained as a colorless solid; R_f_ = 0.76 (SiO_2_, CHCl_3_/MeOH, 9:1); m.p. = 74.5 °C; ${\left[\alpha \right]}_{D}^{20}$ = −62.8° (*c* = 0.12, CHCl_3_); IR (ATR): ν = 3389*w*, 2923*m*, 2847*m*, 1733*s*, 1641*s*, 1609*w*, 1513*s*, 1453*s*, 1402*w*, 1374*w*, 1352*w*, 1316*w*, 1239*m*, 1187*w*, 1134*w*, 1109*w*, 1088*w*, 1028*w*, 1012*w*, 976*w*, 928*w*, 875*w*, 754*m*, 697*m*, 665*w*, 589*w*, 569*w*, 507*w*, 455*w* cm^−1^; ^1^H NMR (400 MHz, CDCl_3_): δ = 7.26 − 6.98 (m, 4H, 23-H, 24-H, 25-H, 27-H), 5.82 (t, *J* = 5.5 Hz, 1H, N-H), 4.41 − 4.31 (m, 2H, 21-H), 2.63 (dd, *J* = 18.7, 3.8 Hz, 1H, 15-H), 2.34 (s, 3H, 28-H), 2.07 − 1.99 (m, 1H, 6-H), 1.99 − 1.91 (m, 1H, 3-H), 1.87 − 1.57 (m, 7H, 1-H, 2-H, 6-H, 7-H, 11-H, 12-H, 15-H), 1.54 (dd, *J* = 11.6, 2.7 Hz, 1H, 14-H), 1.52 − 1.32 (m, 4H, 2-H, 7-H, 12-H, 14-H), 1.21 (s, 3H, 20-H), 1.31 − 1.13 (m, 3H, 3-H, 9-H, 11-H), 1.13 (dd, *J* = 12.1, 2.3 Hz, 1H, 5-H), 0.97 (s, 3H, 17-H), 0.95 − 0.87 (m, 1H, 1-H), 0.76 (s, 3H, 19-H) ppm; ^13^C NMR (101 MHz, CDCl_3_): δ = 222.49 (C-16), 176.47 (C-18), 138.59 (C-22), 138.55 (C-26), 128.94 (C-24), 128.77 (C-27), 128.36 (C-25), 125.06 (C-23), 57.79 (C-5), 54.92 (C-9), 54.44 (C-14), 48.85 (C-13), 48.54 (C-15), 43.86 (C-21), 43.83 (C-4), 41.87 (C-7), 40.33 (C-1), 39.64 (C-10), 38.29 (C-3), 38.25 (C-8), 37.45 (C-12), 30.36 (C-20), 22.42 (C-6), 21.54 (C-28), 20.49 (C-11), 20.00 (C-17), 19.37 (C-2), 13.74 (C-19) ppm; MS (ESI, MeOH/CHCl_3_, 4:1): *m/z* (%) = 420 (100%, [M-H]^−^); analysis calcd for C28H39NO2 (421.63): C 79.76, H 9.32, N 3.32; found: C 79.58, H 9.47, N 3.14.

### 3.23. N-(4-Methylbenzyl)-16-oxostachan-18-amide (20)

Following GPA from **1** (460 mg, 1.44 mmol), 4-methyl-benzylamine (0.74 mL, 5.8 mmol), and chromatography (SiO_2_, CHCl_3_/MeOH, 9:1), **20** (510 mg, 84%) was obtained as a colorless solid; R_f_ = 0.8 (SiO_2_, CHCl_3_/MeOH, 9:1); m.p. = 76 °C; ${\left[\alpha \right]}_{D}^{20}$ = −71.55° (*c* = 0.06, CHCl_3_); IR (ATR): ν = 3388*w*, 2923*m*, 2847*m*, 1734*s*, 1642*s*, 1514*s*, 1453*s*, 1318*w*, 1240*m*, 1182*m*, 1109*w*, 1008*w*, 976*w*, 814*w*, 749*m*, 695*w*, 586*w*, 506*w*, 473*m* cm^−1^; ^1^H NMR (500 MHz, CDCl_3_): δ = 7.20 − 7.06 (m, 4H, 23-H, 24-H, 26-H, 27-H), 5.80 (t, *J* = 5.5 Hz, 1H, N-H), 4.44 − 4.30 (m, 2H, 21-H), 2.64 (dd, *J* = 18.6, 3.8 Hz, 1H, 15-H), 2.34 (s, 3H, 28-H), 2.08 − 1.99 (m, 1H, 3-H), 1.99 − 1.90 (m, 1H, 6-H), 1.85 − 1.70 (m, 4H, 1-H, 2-H, 6-H, 15-H), 1.67 (dt, *J* = 13.2, 3.4 Hz, 2H, 7-H, 11-H), 1.63 − 1.57 (m, 1H, 12-H), 1.54 (dd, *J* = 11.6, 2.8 Hz, 1H, 14-H), 1.50 − 1.31 (m, 4H, 2-H, 7-H, 12-H, 14-H), 1.21 (s, 3H, 20-H), 1.29 − 1.13 (m, 3H, 3-H, 9-H, 11-H), 1.13 (dd, *J* = 12.1, 2.1 Hz, 1H, 5-H), 0.97 (s, 3H, 17-H), 0.92 (td, *J* = 13.2, 4.5 Hz, 1H, 1-H), 0.76 (s, 3H, 19-H) ppm; ^13^C NMR (126 MHz, CDCl_3_): δ = 222.53 (C-16), 176.44 (C-18), 137.34 (C-22), 135.59 (C-25), 129.54 (C-24, C-26), 128.12 (C-23, C-27), 57.79 (C-5), 54.91 (C-9), 54.44 (C-14), 48.86 (C-13), 48.54 (C-15), 43.84 (C-4), 43.63 (C-21), 41.87 (C-7), 40.33 (C-1), 39.65 (C-8), 38.28 (C-3), 38.25 (C-10), 37.46 (C-12), 30.35 (C-20), 22.41 (C-6), 21.25 (C-28), 20.49 (C-11), 20.00 (C-17), 19.38 (C-2), 13.77 (C-19) ppm; MS (ESI, MeOH/CHCl_3_ 4:1): *m/z* (%) = 420 (100%, [M-H]^−^); analysis calcd for C_28_H_39_NO_2_ (421.63): C 79.63, H 9.32, N 3.32; found: C 79.42, H 9.50, N 3.06.

### 3.24. N-(2-Methoxybenzyl)-16-oxostachan-18-amide (21)

Following GPA from **1** (460 mg, 1.44 mmol), 2-methoxy-benzylamine (0.75 mL, 5.76 mmol), and chromatography (SiO_2_, hexanes/ethyl acetate, 95:5), **21** (529 mg, 84%) was obtained as a colorless solid; R_f_ = 0.87 (SiO_2_, CHCl_3_/MeOH, 9:1); m.p. = 56 °C; ${\left[\alpha \right]}_{D}^{20}$ = −64.66° (*c* = 0.115, MeOH); UV-Vis (MeOH): λ_max_ (log ε) = 270 nm (0.09); IR (ATR): ν = 3392*w*, 2924*m*, 2846*m*, 1734*s*, 1648*m*, 1602*w*, 1492*s*, 1457*m*, 1401*w*, 1370*w*, 1317*w*, 1289*w*, 1240*s*, 1171*w*, 1112*m*, 1043*w*, 1028*m*, 976*w*, 929*w*, 855*w*, 815*w*, 750*s*, 696*w*, 665*w*, 615*w*, 586*w*, 531*w*, 507*w*, 490*w* cm^−1^; ^1^H NMR (500 MHz, CDCl_3_): δ = 7.30 − 7.19 (m, 2H, 23-H, 25-H), 6.95 − 6.83 (m, 2H, 24-H, 26-H), 6.21 (t, *J* = 5.7 Hz, 1H, N-H), 4.40 − 4.33 (m, 2H, 21-H), 3.85 (s, 3H, 28-H), 2.54 (dd, *J* = 18.6, 3.8 Hz, 1H, 15-H), 2.08 − 1.98 (m, 1H, 3-H), 1.96 − 1.89 (m, 1H, 6-H), 1.81 − 1.59 (m, 6H, 1-H, 2-H, 6-H, 7-H, 11-H, 15-H), 1.59 − 1.54 (m, 1H, 12-H), 1.53 − 1.49 (m, 1H, 14-H), 1.49 − 1.29 (m, 4H, 2-H, 7-H, 12-H, 14-H), 1.16 (s, 3H, 20-H), 1.15 − 1.10 (m, 3H, 3-H, 9-H, 11-H), 1.09 − 1.05 (m, 1H, 5-H), 0.95 (s, 3H, 17-H), 0.88 (td, *J* = 13.1, 4.4 Hz, 1H, 1-H), 0.51 (s, 3H, 19-H) ppm; ^13^C NMR (126 MHz, CDCl_3_): δ = 222.51 (C-16), 176.27 (C-18), 157.63 (C-27), 130.56 (C-25), 129.00 (C-23), 126.30 (C-22), 120.87 (C-24), 110.21 (C-26), 57.72 (C-5), 55.30 (C-28), 54.83 (C-9), 54.39 (C-14), 48.80 (C-13), 48.51 (C-15), 43.74 (C-4), 41.90 (C-7), 40.32 (C-1), 40.00 (C-21), 39.57 (C-8), 38.19 (C-3), 38.11 (C-10), 37.41 (C-12), 30.19 (C-20), 22.18 (C-6), 20.41 (C-11), 19.97 (C-17), 19.08 (C-2), 13.07 (C-19) ppm; MS (ESI, MeOH/CHCl_3_, 4:1): *m/z* (%) = 438 (90%, [M+H]^+^), 897 (100%, [2M+Na]^+^); analysis calcd for C_28_H_39_NO_3_ (437.62): C 76.85, H 8.98, N 3.20; found: C 76.60, H 9.22, N 2.97.

### 3.25. N-(3-Methoxybenzyl)-16-oxostachan-18-amide (22)

Following GPA from **1** (500 mg, 1.57 mmol), 3-methoxy-benzylamine (0.82 mL, 6.28 mmol), and chromatography (SiO_2_, hexanes/ethyl acetate, 95:5), **22** (498 mg, 71%) was obtained as a colorless solid; R_f_ = 0.89 (SiO_2_, CHCl_3_/MeOH, 9:1); m.p. = 58.5 °C; ${\left[\alpha \right]}_{D}^{20}$ = −56.57° (*c* = 0.069, MeOH); UV-Vis (MeOH): λ_max_ (log ε) = 273 nm (0.17); IR (ATR): ν = 3390*w*, 2925*m*, 2846*m*, 1733*s*, 1650*m*, 1601*m*, 1586*m*, 1507*s*, 1489*s*, 1454*s*, 1352*w*, 1316*w*, 1262*s*, 1189*w*, 1151*m*, 1110*w*, 1087*w*, 1043*m*, 1008*w*, 976*w*, 874*w*, 854*w*, 776*m*, 738*m*, 694*m*, 569*w*, 554*w*, 507*w*, 465*w* cm^−1^; ^1^H NMR (400 MHz, CDCl_3_): δ = 7.28 − 7.21 (m, 1H, 24-H), 6.90 − 6.79 (m, 3H, 23-H, 25-H, 27-H), 5.85 (t, *J* = 5.6 Hz, 1H, N-H), 4.42 − 4.29 (m, 2H, 21-H), 3.79 (s, 3H, 28-H), 2.63 (dd, *J* = 18.6, 3.8 Hz, 1H, 15-H), 2.07 − 1.99 (m, 1H, 3-H), 1.99 − 1.91 (m, 1H, 6-H), 1.87 − 1.64 (m, 6H, 1-H, 2-H, 6-H, 7-H, 11-H, 15-H), 1.63 − 1.57 (m, 1H, 12-H), 1.54 (dd, *J* = 11.6, 2.7 Hz, 1H, 14-H), 1.51 − 1.31 (m, 4H, 2-H, 7-H, 12-H, 14-H), 1.22 (s, 3H, 20-H), 1.19 (s, 3H, 3-H, 9-H, 11-H), 1.13 (m, 1H, 5-H), 0.97 (s, 3H, 17-H), 0.94 − 0.88 (m, 1H, 1-H), 0.76 (s, 3H, 19-H) ppm; ^13^C NMR (101 MHz, CDCl_3_): δ = 222.48 (C-16), 176.50 (C-18), 160.04 (C-26), 140.26 (C-22), 129.89 (C-24), 120.27 (C-23), 113.62 (C-25), 113.10 (C-27), 57.77 (C-5), 55.41 (C-28), 54.91 (C-9), 54.42 (C-14), 48.84 (C-13), 48.53 (C-15), 43.89 (C-4), 43.77 (C-21), 41.85 (C-7), 40.32 (C-1), 39.63 (C-8), 38.28 (C-3), 38.24 (C-10), 37.44 (C-12), 30.36 (C-20), 22.41 (C-6), 20.48 (C-11), 19.99 (C-17), 19.37 (C-2), 13.77 (C-19) ppm; MS (ESI, MeOH/CHCl_3_ 4:1): *m/z* (%) = 436 (100%, [M-H]^−^); analysis calcd for C_28_H_39_NO_3_ (437.62): C 76.85, H 8.98, N 3.20; found: C 76.58, H 9.13, N 3.07.

### 3.26. N-(4-Methoxybenzyl)-16-oxostachan-18-amide (23)

Following GPA from **1** (500 mg, 1.57 mmol), 3-methoxy-benzylamine (0.61 mL, 4.71 mmol), and chromatography (SiO_2_, hexanes/ethyl acetate, 95:5), **23** (618 mg, 90%) was obtained as a colorless solid; R_f_ = 0.9 (SiO_2_, CHCl_3_/MeOH, 9:1); m.p. = 59 °C; ${\left[\alpha \right]}_{D}^{20}$ = −59.89° (*c* = 0.1, MeOH); UV-Vis (MeOH): λ_max_ (log ε) = 224 nm (0.66); IR (ATR): ν = 3396*w*, 2925*m*, 2846*m*, 1733*m*, 1642*m*, 1612*w*, 1511*s*, 1453*m*, 1356*w*, 1317*w*, 1301*w*, 1245*s*, 1179*m*, 1159*w*, 1109*w*, 1087*w*, 1032*m*, 976*w*, 929*w*, 828*m*, 751*m*, 696*w*, 665*w*, 588*w*, 563*w*, 508*m*, 415*w* cm^−1^; ^1^H NMR (400 MHz, CDCl_3_): δ = 7.20 − 7.13 (m, 2H, 23-H, 27-H), 6.88 − 6.82 (m, 2H, 24-H, 26-H), 5.79 (t, *J* = 5.0 Hz, 1H, N-H), 4.40 − 4.26 (m, 2H, 21-H), 3.80 (s, 3H, 28-H), 2.63 (dd, *J* = 18.7, 3.8 Hz, 1H, 15-H), 2.05 − 1.98 (m, 1H, 3-H), 1.98 − 1.91 (m, 1H, 6-H), 1.85 − 1.64 (m, 6H, 1-H, 2-H, 6-H, 7-H, 11-H, 15-H), 1.63 − 1.57 (m, 1H, 12-H), 1.54 (dd, *J* = 11.6, 2.7 Hz, 1H, 14-H), 1.42 (m, 4H, 2-H, 7-H, 12-H, 14-H), 1.29 − 1.13 (m, 3H, 3-H, 9-H, 11-H), 1.20 (s, 3H, 20-H), 1.12 (m, 1H, 5-H), 0.97 (s, 3H, 17-H), 0.95 − 0.86 (m, 1H, 1-H), 0.75 (s, 3H, 19-H) ppm; ^13^C NMR (101 MHz, CDCl_3_): δ = 222.53 (C-16), 176.41 (C-18), 159.16 (C-25), 130.71 (C-22), 129.44 (C-23, C-27), 114.24 (C-24, C-26), 57.78 (C-5), 55.44 (C-28), 54.89 (C-9), 54.42 (C-14), 48.85 (C-13), 48.53 (C-15), 43.82 (C-4), 43.31 (C-21), 41.85 (C-7), 40.3(C-1), 39.64 (C-8) 38.24 (C-3, C-10), 37.45 (C-12), 30.33 (C-20), 22.40 (C-6), 20.48 (C-11), 19.99 (C-17), 19.36 (C-2), 13.76 (C-19) ppm; MS (ESI, MeOH/CHCl_3_ 4:1): *m/z* (%) = 436 (100%, [M-H]^−^); analysis calcd for C_28_H_39_NO_3_ (437.62): C 76.85, H 8.98, N 3.20; found: C 76.63, H 9.17, N 3.01.

### 3.27. 18-Oxo-18-piperazin-1-yl-stachan-16-one (24)

Following GPA from **1** (2.0 g, 6.28 mmol), piperazine (3.1 g, 36 mmol), and chromatography (SiO_2_, CHCl_3_/MeOH, 95:5), **24** (2.28 g, 94%) was obtained as a colorless solid; R_f_ = 0.5 (SiO_2_, CHCl_3_/MeOH, 9:1); m.p. = 137 °C; ${\left[\alpha \right]}_{D}^{20}$ = −26.12° (*c* = 0.117, CHCl_3_); IR (ATR): ν = 2855*w*, 1726*s*, 1640*s*, 1453*m*, 1397*m*, 1328*w*, 1313*w*, 1252*w*, 1220*w*, 1177*m*, 1142*w*, 1108*w*, 1056*w*, 1030*m*, 978*w*, 796*m*, 590*w*, 556*w*, 519*w*, 505*w* cm^−1^; ^1^H NMR (500 MHz, CDCl_3_): δ = 4.28 (*s*, 1H, N-H), 3.72 − 3.57 (*m*, 4H, 21-H, 24-H), 2.99 − 2.86 (*m*, 4H, 22-H, 23-H), 2.72 (*dd*, *J* = 18.7, 3.8 Hz, 1H, 15-H), 2.28 (*dd*, *J* = 14.2, 3.7 Hz, 1H, 3-H), 2.16 − 2.04 (*m*, 1H, 6-H), 1.83 − 1.75 (*m*, 2H, 6-H, 15-H), 1.71 − 1.62 (*m*, 3H, 1-H, 7-H, 11-H), 1.61 − 1.57 (*m*, 1H, 12-H), 1.55 (*s*, 1H, 2-H), 1.53 (*dd*, *J* = 11.6, 2.8 Hz, 1H, 14-H), 1.45 (*dt*, *J* = 6.4, 2.8 Hz, 1H, 2-H), 1.41 (*dd*, *J* = 11.3, 3.7 Hz, 1H, 7-H), 1.42 − 1.36 (*m*, 1H, 14-H), 1.34 (*dd*, *J* = 12.3, 5.2 Hz, 1H, 12-H), 1.28 (*s*, 3H, 20-H), 1.24 (*td*, *J* = 12.9, 4.9 Hz, 1H, 11-H), 1.20 − 1.11 (*m*, 2H, 3-H, 9-H), 1.01 (*dd*, *J* = 11.8, 1.8 Hz, 1H, 5-H), 0.96 (*s*, 3H, 17-H), 0.91 (*td*, *J* = 13.2, 4.3 Hz, 1H, 1-H), 0.82 (*s*, 3H, 19-H) ppm; ^13^C NMR (126 MHz, CDCl_3_): δ = 222.79 (C-16), 176.61 (C-18), 61.99 (C-5), 56.23 (C-9), 54.57 (C-14), 48.84 (C-13), 48.68 (C-15), 46.96 (C-22), 46.28 (C-4), 46.13 (C-21), 45.88 (C-25), 45.59 (C-24), 42.60 (C-7), 40.90 (C-1), 39.81 (C-3), 39.78 (C-8), 38.73 (C-10), 37.49 (C-12), 28.14 (C-20), 22.64 (C-6), 20.54 (C-11), 20.02 (C-17), 19.99 (C-2), 16.19 (C-19) ppm; MS (ESI, MeOH/CHCl_3_, 4:1): *m/z* (%) = 387 (100%, [M+H]^+^), 795 (30%, [2M+Na]^+^); analysis calcd for C_24_H_38_N_2_O_2_ (386.58): C 74.57, H 9.91, N 7.23; found: C 74.41, H 10.08, N 7.02.

### 3.28. 18-(1,4-Diazepan-1-yl)-18-oxostachan-16-one (25)

Following GPA from **1** (2.0 g, 6.28 mmol), homopiperazine (2.52 g, 36 mmol), and chromatography (SiO_2_, CHCl_3_/MeOH, 95:5), **25** (1.78 g, 71%) was obtained as a colorless solid; R_f_ = 0.4 (SiO_2_, CHCl_3_/MeOH, 9:1); m.p. = 162 °C; ${\left[\alpha \right]}_{D}^{20}$ = −17.37° (*c* = 0.019, CHCl_3_); IR (ATR): ν = 2922*m*, 1735*s*, 1628*s*, 1458*m*, 1401*m*, 1172*m* cm^−1^; ^1^H NMR (500 MHz, CDCl_3_): δ = 5.29 (*s*, 1H, N-H), 3.74 − 3.57 (*m*, 4H, 21-H, 25-H), 3.14 − 2.86 (*m*, 4H, 22-H, 23-H), 2.76 − 2.64 (*m*, 1H, 15-H), 2.34 (*td*, *J* = 14.5, 3.5 Hz, 1H, 3-H), 2.18 − 2.08 (*m*, 1H, 6-H), 1.97 (*ttt*, *J* = 12.8, 8.9, 4.0 Hz, 2H, 24-H), 1.87 − 1.75 (*m*, 2H, 6-H, 15-H), 1.73 − 1.56 (*m*, 5H, 1-H, 2-H, 7-H, 11-H, 12-H), 1.53 (*dd*, *J* = 11.5, 2.7 Hz, 1H, 14-H), 1.50 − 1.32 (*m*, 4H, 2-H, 7-H, 11-H, 12.H), 1.29 (*s*, 3H, 20-H), 1.28 − 1.10 (*m*, 3H, 3-H, 9-H, 11-H), 1.01 (*dd*, *J* = 11.8, 1.9 Hz, 1H, 5-H), 0.96 (*s*, 3H, 17-H), 0.95 − 0.90 (*m*, 1H, 1-H), 0.83 (*s*, 3H, 19-H) ppm; ^13^C NMR (126 MHz, CDCl_3_): δ = 222.84 (C-16), 176.77 (C-18), 62.63 (C-5), 56.25 (C-9), 54.58 (C-14), 50.16 (C-26), 48.85 (C-13), 48.72 (C-22), 48.66 (C-15), 47.91 (C-21), 46.85 (C-4), 46.61 (C-23), 42.66 (C-7), 41.06 (C-1), 39.85 (C-3), 39.49 (C-8), 38.78 (C-10), 37.51 (C-12), 28.79 (C-25), 28.35 (C-20), 22.89 (C-6), 20.58 (C-11), 20.17 (C-17), 20.03 (C-2), 16.22 (C-19) ppm; MS (ESI, MeOH/CHCl_3_, 4:1): *m/z* (%) = 401 (100%, [M+H]^+^); analysis calcd for C_25_H_40_N_2_O_2_ (400.61): C 74.96, H 10.06, N 6.99; found: C 74.70, H 10.24, N 6.72.

### 3.29. 18-Morpholin-4-yl-18-oxostachan-16-one (26)

Following GPA from **1** (270 mg, 0.85 mmol), morpholine (0.44 mL, 5.1 mmol), and chromatography (SiO_2_, CHCl_3_/MeOH, 95:5), **26** (247 mg, 75%) was obtained as a colorless solid; R_f_ = 0.51 (SiO_2_, CHCl_3_/MeOH, 9:1); m.p. = 155 °C; ${\left[\alpha \right]}_{D}^{20}$ = −27.15° (*c* = 0.11, CHCl_3_); IR (ATR): ν = 2922*m*, 2850*m*, 1733*s*, 1644*m*, 1452*m*, 1389*m*, 1373*m*, 1264*m*, 1224*m*, 1174*m*, 1111*s*, 1068*w*, 1027*m*, 977*w*, 928*w*, 892*w*, 844*w*, 752*w*, 694*w*, 605*w*, 508*w* cm^−1^; ^1^H NMR (500 MHz, CDCl_3_): δ = 3.75 − 3.48 (*m*, 8H, 21-H, 22-H, 23-H, 24-H), 2.73 (*dd*, *J* = 18.7, 3.8 Hz, 1H, 15-H), 2.29 (*dt*, *J* = 15.1, 3.5 Hz, 1H, 3-H), 2.10 (*td*, *J* = 13.9, 3.2 Hz, 1H, 6-H), 1.91 − 1.76 (*m*, 2H, 6-H, 15-H), 1.71 − 1.62 (*m*, 3H, 1-H, 7-H, 11-H), 1.62 − 1.56 (*m*, 2H, 2-H, 12-H), 1.53 (*dd*, *J* = 11.6, 2.8 Hz, 1H, 14-H), 1.49 − 1.41 (*m*, 2H, 2-H, 7-H), 1.41 − 1.32 (*m*, 2H, 12-H, 14-H), 1.28 (*s*, 3H, 20-H), 1.24 (*s*, 1H, 11-H), 1.21 − 1.15 (*m*, 2H, 3-H, 9-H), 1.01 (*dd*, *J* = 11.8, 1.9 Hz, 1H, 5-H), 0.96 (*s*, 3H, 17-H), 0.94 − 0.88 (*m*, 1H, 1-H), 0.84 (*s*, 3H, 19-H) ppm; ^13^C NMR (126 MHz, CDCl_3_): δ = 222.80 (C-16), 176.63 (C-18), 67.11 (C-22, C-23), 61.96 (C-5), 56.23 (C-9), 54.59 (C-14), 48.85 (C-13), 48.68 (C-15), 46.84 (C-21, C-24), 46.27 (C-4), 42.61 (C-7), 40.93 (C-1), 39.82 (C-8), 39.76 (C-3), 38.73 (C-10), 37.50 (C-12), 28.15 (C-20), 22.67 (C-6), 20.56 (C-11), 20.02 (C-17), 19.98 (C-2), 16.17 (C-19) ppm; MS (ESI, MeOH/CHCl_3_, 4:1): *m/z* (%) = 388 (100%, [M+H]^+^); analysis calcd for C_24_H_37_NO_3_ (387.56): C 74.77, H 9.79, N 3.49; found: C 74.50, H 9.97, N 3.28.

### 3.30. 18-(1,4-Oxazepan-4-yl)-18-oxostachan-16-one (27)

Following GPA from **1** (250 mg, 0.785 mmol), homomorpholine (166 mg, 1.21 mmol), and chromatography (SiO_2_, CHCl_3_/MeOH, 95:5), **27** (195 mg, 62%) was obtained as a colorless solid; R_f_ = 0.65 (SiO_2_, CHCl_3_/MeOH, 9:1); m.p. = 229 °C; ${\left[\alpha \right]}_{D}^{20}$ = −11.26° (*c* = 0.091, CHCl_3_); IR (ATR): ν = 2922*m*, 2845*m*, 1736*s*, 1626*s*, 1459*m*, 1404*w*, 1363*w*, 1252*w*, 1219*w*, 1183*w*, 1123*s*, 1013*w*, 976*w*, 963*w*, 931*w*, 849*w*, 751*w*, 696*w*, 574*w*, 507*w* cm^−1^; ^1^H NMR (500 MHz, CDCl_3_): δ = 3.85 − 3.56 (*m*, 8H, 21-H, 22-H, 23-H, 25-H), 2.71 (*dd*, *J* = 18.7, 3.8 Hz, 1H, 15-H), 2.49 − 2.41 (*m*, 1H, 3-H), 2.34 (*ddt*, *J* = 14.4, 4.1, 2.1 Hz, 1H, 6-H), 2.23 − 2.10 (*m*, 1H, 24-H), 1.99 − 1.88 (*m*, 1H, 24-H), 1.87 − 1.81 (*m*, 1H, 6-H), 1.78 (*d*, *J* = 18.8 Hz, 1H, 15-H), 1.73 − 1.56 (*m*, 5H, 1-H, 2-H, 7-H, 11-H, 12-H), 1.53 (*dd*, *J* = 11.7, 2.6 Hz, 1H, 14-H), 1.50 − 1.42 (*m*, 1H, 2-H), 1.42 − 1.31 (*m*, 3H, 7-H, 12-H, 14-H), 1.29 (*s*, 3H, 20-H), 1.26 (*s*, 1H, 11-H), 1.21 − 1.09 (*m*, 2H, 3-H, 9-H), 1.01 (*dd*, *J* = 11.8, 1.9 Hz, 1H, 5-H), 0.96 (*s*, 3H, 17-H), 0.94 (*s*, 1H, 1-H), 0.84 (*s*, 3H, 19-H) ppm; ^13^C NMR (126 MHz, CDCl_3_): δ = 222.87 (C-16), 176.64 (C-18), 71.05 (C-23), 69.63 (C-22), 62.68 (C-5), 56.26 (C-9), 54.61 (C-14), 51.89 (C-25), 48.85 (C-13), 48.67 (C-15), 47.80 (C-21), 46.85 (C-4), 42.69 (C-7), 41.14 (C-1), 39.86 (C-8), 39.59 (C-3), 38.80 (C-10),37.53 (C-12), 30.51 (C-24), 28.39 (C-20), 22.93 (C-6), 20.59 (C-11), 20.15 (C-2), 20.04 (C-17), 19.90, 16.22 (C-19) ppm; MS (ESI, MeOH/CHCl_3_, 4:1): *m/z* (%) = 402 (85%, [M+H] ^+^), 425 (80%, [M+Na]^+^), 826 (100%, [2M+H]^+^); analysis calcd for C_25_H_39_NO_3_ (401.59): C 74.77, H 9.79, N 3.49; found: C 74.47, H 9.97, N 3.29.

### 3.31. 18-Oxo-18-thiomorpholin-4-yl-stachan-16-one (28)

Following GPA from **1** (270 mg, 0.85 mmol), thiomorpholine (0.52 mL, 5.1 mmol), and chromatography (SiO_2_, CHCl_3_/MeOH, 95:5), **28** (198 mg, 58%) was obtained as a colorless solid; R_f_ = 0.86 (SiO_2_, CHCl_3_/MeOH, 9:1); m.p. = 137 °C; ${\left[\alpha \right]}_{D}^{20}$ = −31.96° (*c* = 0.148, CHCl_3_); IR (ATR): ν = 2921*m*, 2844*m*, 1736*s*, 1639*s*, 1459*m*, 1393*m*, 1359*m*, 1274*w*, 1243*w*, 1215*w*, 1160*s*, 1112*w*, 1025*w*, 959*s*, 848*w*, 758*w*, 695*w*, 604*w*, 496*w* cm^−1^; ^1^H NMR (500 MHz, CDCl_3_): δ = 3.89 − 3.75 (*m*, 4H, 21-H, 24-H), 2.72 (*dd*, *J* = 18.7, 3.8 Hz, 1H, 15-H), 2.68 − 2.50 (*m*, 4H, 22-H, 23-H), 2.28 (*dt*, *J* = 14.0, 3.0 Hz, 1H, 3-H), 2.19 − 2.06 (*m*, 1H, 6-H), 1.79 (*d*, *J* = 18.8 Hz, 1H, 15-H), 1.78 (*s*, 1H, 6-H), 1.71 (*dt*, *J* = 18.8, 2.3 Hz, 1H, 1-H), 1.68 − 1.63 (*m*, 2H, 7-H, 11-H), 1.63 − 1.56 (*m*, 1H, 12-H), 1.53 (*dd*, *J* = 11.5, 2.8 Hz, 1H, 14-H), 1.54 − 1.44 (*m*, 1H, 2-H), 1.47 − 1.38 (*m*, 2H, 7-H, 14-H), 1.37 − 1.32 (*m*, 1H, 12-H), 1.29 (*s*, 3H, 20-H), 1.28 − 1.23 (*m*, 1H, 11-H), 1.17 (*dd*, *J* = 11.5, 3.5 Hz, 2H, 9-H), 1.13 (*d*, *J* = 3.2 Hz, 1H, 3-H), 1.00 (*dd*, *J* = 11.7, 1.6 Hz, 1H, 5-H), 0.96 (*s*, 3H, 17-H), 0.92 (*td*, *J* = 13.1, 4.3 Hz, 1H, 1-H), 0.82 (*s*, 3H, 19-H) ppm; ^13^C NMR (126 MHz, CDCl_3_): δ = 222.84 (C-16), 176.56 (C-18), 62.37 (C-5), 56.31 (C-9), 54.60 (C-14), 48.92 (C-21, C-24), 48.86 (C-13), 48.68 (C-15), 46.46 (C-4), 42.68 (C-7), 41.02 (C-1), 39.85 (C-8), 39.63 (C-3), 38.76 (C-10), 37.51 (C-12), 28.23 (C-20), 27.67 (C-22, C-23), 22.71 (C-6), 20.56 (C-11), 20.07 (C-2), 20.03 (C-17), 16.31 (C-19) ppm; MS (ESI, MeOH/CHCl_3_, 4:1): *m/z* (%) = 404 (100%, [M+H]^+^); analysis calcd for C_24_H_37_NSO_2_ (403.63): C 71.42, H 9.24, N 3.47; found: C 71.25, H 9.40, N 3.28.

### 3.32. 18-Oxo-18-(1,4-thiazepan-4-yl)-stachan-16-one (29)

Following GPA from **1** (270 mg, 0.85 mmol), homothiomorpholine (194 mg, 1.27 mmol), and chromatography (SiO_2_, CHCl_3_/MeOH, 95:5), **29** (245 mg, 69%) was obtained as a colorless solid; R_f_ = 0.5 (SiO_2_, CHCl_3_/MeOH, 9:1); m.p. = 198 °C; ${\left[\alpha \right]}_{D}^{20}$ = −18.43° (*c* = 0.1, CHCl_3_); IR (ATR): ν = 2920*m*, 2843*m*, 1737*s*, 1624*s*, 1462*m*, 1401*m*, 1358*m*, 1274*w*, 1162*m*, 1111*w*, 975*w*, 881*m*, 753*w*, 489*w* cm^−1^; ^1^H NMR (500 MHz, CDCl_3_): δ = 4.00 (*ddd*, *J* = 14.3, 5.5, 4.1 Hz, 1H, 25-H), 3.81 (*dt*, *J* = 14.4, 5.3 Hz, 1H, 21-H), 3.65 − 3.52 (*m*, 2H, 21-H, 25-H), 2.86 − 2.67 (*m*, 4H, 15-H, 22-H, 24-H), 2.61 (*ddd*, *J* = 14.7, 9.4, 5.1 Hz, 1H, 22-H), 2.32 (*dt*, *J* = 12.8, 2.7 Hz, 1H, 3-H), 2.22 − 2.12 (*m*, 1H, 6-H), 2.11 − 2.04 (*m*, 1H, 23-H), 2.00 − 1.90 (*m*, 1H, 23-H), 1.84 (*dt*, *J* = 13.7, 2.9 Hz, 1H, 6-H), 1.79 (*d*, *J* = 18.7 Hz, 1H, 15-H), 1.76 − 1.69 (*m*, 1H, 1-H), 1.69 − 1.55 (*m*, 4H, 2-H, 7-H, 11-H, 12-H), 1.53 (*dd*, *J* = 11.6, 2.8 Hz, 1H, 14-H), 1.50 − 1.46 (*m*, 1H, 2-H), 1.45 -1.34 (*m*, 2H, 7-H, 14-H), 1.36 − 1.21 (*m*, 2H, 11-H, 12-H), 1.29 (*s*, 3H, 20-H), 1.21 − 1.13 (*m*, 2H, 3-H, 9-H), 1.00 (*dd*, *J* = 11.8, 1.9 Hz, 1H, 5-H), 0.96 (*s*, 3H, 17-H), 0.93 (*d*, *J* = 4.2 Hz, 1H, 1-H), 0.86 − 0.83 (*m*, 3H, 19-H) ppm; ^13^C NMR (126 MHz, CDCl_3_): δ = 222.91 (C-16), 176.43 (C-18), 62.82 (C-5), 56.25 (C-9), 54.61 (C-14), 53.95 (C-25), 49.50 (C-21), 48.88 (C-15), 48.68 (C-13), 47.01 (C-4), 42.73 (C-7), 41.16 (C-1), 39.89 (C-8), 39.27 (C-3), 38.82 (C-10), 37.55 (C-12), 34.32 (C-24), 31.88 (C-22), 30.37 (C-23), 28.55 (C-20), 22.98 (C-6), 20.62 (C-11), 20.45 (C-2), 20.06 (C-17), 16.23 (C-19) ppm; MS (ESI, MeOH/CHCl_3_, 4:1): *m/z* (%) = 419 (100%, [M+H]^+^), 859 (67%, [2M+Na]^+^); analysis calcd for C_25_H_39_NSO_2_ (417.65): C 71.90, H 9.41, N 3.35; found: C 71.70, H 9.65, N 3.16.

### 3.33. N-(2-Aminoethyl)-16-oxostachan-18-amide (30)

Following GPA from **1** (270 mg, 0.85 mmol), ethylenediamine (0.34 mL, 5.1 mmol), and chromatography (SiO_2_, CHCl_3_/MeOH, 95:5), **30** (270 mg, 88%) was obtained as a colorless solid; R_f_ = 0.25 (SiO_2_, CHCl_3_/MeOH, 9:1); m.p. = 105 °C; ${\left[\alpha \right]}_{D}^{20}$ = −71.24° (*c* = 0.121, CHCl_3_); IR (ATR): ν = 2924*s*, 1735*s*, 1637*s*, 1516*s*, 1453*s* cm^−1^; ^1^H NMR (500 MHz, CDCl_3_): δ = 8.21 − 8.16 (*m*, 1H, NH), 6.75 − 6.70 (*m*, 2H, NH_2_), 3.57 − 3.27 (*m*, 2H, 22-H), 3.23 (*s*, 2H, 21-H), 2.66 − 2.55 (*m*, 1H, 15-H), 2.18 − 2.09 (*m*, 1H, 3-H,), 2.05 (*d*, *J* = 16.2 Hz, 1H, 6-H), 1.80 − 1.64 (*m*, 5H, 1-H, 2-H, 7-H, 11-H, 12-H), 1.46 − 1.45 (*dd*, 1H, 14-H), 1.36 − 1.22 (*m*, 4H, 2-H, 7-H, 11-H, 12-H), 1.17 (*s*, 3H, 20-H), 1.15 (*m*, 1H, 9-H, 11-H), 1.12 − 1.06 (*m*, 1H, 5-H), 0.96 (*d*, *J* = 1.4 Hz, 3H, 17-H), 0.91 − 0.82 (*m*, 1H, 1-H), 0.80 (*s*, 3H, 19-H) ppm; ^13^C NMR (126 MHz, CDCl_3_): δ = 222.57 (C-16), 178.63 (C-18), 139.92 (C-22), 106.67 (C-23), 57.48 (C-5), 55.57 (C-9), 54.76 (C-14), 50.06 (C-13), 48.70 (C-15), 48.47 (C-4), 43.84 (C-7), 40.16 (C-1), 40.09 (C-3), 39.51 (C-8), 38.17 (C-10), 37.29 (C-12), 29.96 (C-20), 22.12 (C-6), 20.33 (C-11), 19.83 (C-17), 19.13 (C-2), 13.54 (C-19) ppm; MS (ESI, MeOH/CHCl_3_, 4:1): *m/z* (%) = 359 (100%, [M-H]^−^); analysis calcd for C_22_H_36_N_2_O_2_ (360.54): C 73.29, H 10.06, N 7.77; found: C 72.96, H 10.30, N 7.49.

### 3.34. 18-(4-{2-[3,6-Bis(diethylamino)xanthenium-9-yl]-benzoyl}-piperazin-1-yl)-18-oxostachan-16-one Chloride (31)

Following GPC from **24** (400 mg, 1.03 mmol), oxalyl chloride (0.35 mL, 4.14 mmol), rhodamine B (744 mg, 1.55 mmol), NEt_3_ (0.16 mL, 1.14 mmol), and chromatography (SiO_2_, CHCl_3_/MeOH, 95:5), **31** (410 mg, 47%) was obtained as a purple solid; R_f_ = 0.3 (SiO_2_, CHCl_3_/MeOH, 9:1); m.p. = 193 °C; UV-Vis (MeOH): λ_max_ (log ε) = 561.85 nm (1.74); IR (ATR): ν = 2924*w*, 1733*w*, 1632*w*, 1585*s*, 1528*w*, 1451*m*, 1411*m*, 1335*s*, 1272*m*, 1247*m*, 1179*s*, 1132*m*, 1072*m*, 1003*m*, 976*w*, 922*w*, 824*w*, 747*m*, 683*m*, 498*w* cm^−1^; ^1^H NMR (500 MHz, CDCl_3_): δ = 7.75 − 7.27 (*m*, 6H, 28-H, 29-H, 30-H, 31-H, 35-H, 38-H), 7.12 − 6.98 (*m*, 2H, 37-H), 6.78 (*s*, 2H, 35-H), 3.88 − 3.14 (*m*, 16H, 21-H, 22-H, 23-H, 24-H, 39-H), 2.66 (*dd*, *J* = 18.6, 3.7 Hz, 1H, 15-H), 2.29 − 2.22 (*m*, 1H, 3-H), 2.07 − 1.96 (*m*, 1H, 6-H), 1.80 − 1.72 (*m*, 2H, 6-H, 15-H), 1.70 − 1.54 (*m*, 4H, 1-H, 7-H, 11-H, 12-H), 1.51 (*dd*, *J* = 11.7, 2.6 Hz, 1H, 14-H), 1.47 − 1.08 (*m*, 11H, 2-H, 3-H, 7-H, 9-H, 11-H, 12-H, 14-H, 20-H), 0.98 (*d*, *J* = 11.8 Hz, 1H, 5-H), 0.96 − 0.86 (*m*, 15H, 17-H, 40-H), 0.84 − 0.81 (*m*, 1H, 1-H), 0.75 (*s*, 3H, 19-H) ppm; ^13^C NMR (126 MHz, CDCl_3_): δ = 222.87 (C-16), 177.31 (C-18), 167.85 (C-25), 157.86 (C-36), 155.94 (C-32), 155.77 (C-34), 135.24 (C-27), 132.58 (C-26), 132.39 (C-28), 130.98 (C-38), 130.84 (C-33), 130.44 (C-29), 130.38 (C-31), 130.28 (C-30), 114.55 (C-37), 96.50 (C-35), 61.81 (C-5), 56.02 (C-9), 54.47 (C-14), 48.83 (C-13), 48.66 (C-15), 46.36 (C-21, C-24), 46.33 (C-22, C-23), 46.28 (C-4), 42.48 (C-7), 42.16 (C-39), 40.78 (C-1), 39.78 (C-8), 39.51 (C-3), 38.65 (C-10), 37.44 (C-12), 28.09 (C-20), 22.54 (C-6), 20.50 (C-11), 19.98 (C-17), 19.88 (C-2), 12.82 (C-40), 11.07 (C-19) ppm; MS (ESI, MeOH/CHCl_3_, 4:1): *m/z* (%) = 812 (78%, [M-Cl]^+^); analysis calcd for C_52_H_67_N_4_O_4_Cl (847.58): C 73.69, H 7.97, N 6.61; found: C 73.40, H 8.16, N 6.45.

### 3.35. 18-(4-{2-[3,6-Bis(diethylamino)xanthenium-9-yl]-benzoyl}-1,4-diazepan-1-yl)-18-oxostachan-16-one Chloride (32)

Following GPC from **25** (200 mg, 0.49 mmol), oxalyl chloride (0.17 mL, (2.0 mmol), rhodamine B (359.3 mg, 0.75 mmol), NEt3 (0.1 mL, 0.05 mmol), and chromatography (SiO_2_, CHCl_3_/MeOH, 95:5), **32** (207 mg, 49%) was obtained as a purple solid; R_f_ = 0.2 (SiO_2_, CHCl_3_/MeOH, 9:1); m.p. = 185 °C; UV-Vis (MeOH): λ_max_ (log ε) = 561.92 nm (2.55); IR (ATR): ν = 2925*w*, 1734*w*, 1586*s*, 1528*w*, 1466*m*, 1412*w*, 1336*s*, 1274*m*, 1246*m*, 1179*s*, 1132*m*, 1073*m*, 1011*w*, 976*w*, 920*w*, 822*w*, 771*m*, 682*m*, 619*w*, 577*w*, 497*w* cm^−1^; ^1^H NMR (500 MHz, CDCl_3_): δ = 7.68 − 7.22 (*m*, 6H, 29-H, 30-H, 31-H, 32-H, 39-H), 6.98 − 6.74 (*m*, 4H, 38-H, 39-H), 3.98 − 3.11 (*m*, 16H, 21-H, 22-H, 23-H, 25-H, 40-H), 2.76 − 2.64 (*m*, 1H, 15-H), 2.31 (*d*, *J* = 14.4 Hz, 1H, 3-H), 2.19 − 1.97 (*m*, 1H, 6-H), 1.91 − 1.73 (*m*, 4H, 6-H, 15-H, 24-H), 1.72 − 1.54 (*m*, 4H, 1-H, 7-H, 11-H, 12-H), 1.55 − 1.05 (*m*, 24H, 2-H, 3-H, 7-H, 9-H, 11-H, 12-H, 14-H, 20-H, 41-H), 1.06 − 0.74 (*m*, 8H, 1-H, 5-H, 17-H, 19-H) ppm; ^13^C NMR (126 MHz, CDCl_3_): δ = 222.94 (C-16), 176.96 (C-18), 168.99 (C-26), 168.49 (C-37), 157.93 (C-33), 155.78 (C-35), 140.56 (C-39), 136.52 (C-28), 136.30 (C-27), 132.65 (C-29), 132.43 (C-30), 129.57 (C-32), 126.87 (C-31), 114.09 (C-38), 113.73 (C-34), 96.90 (C-36), 62.25 (C-5), 56.21 (C-9), 54.56 (C-14), 50.49 (C-25), 49.83 (C-23), 48.86 (C-13), 48.65 (C-15), 46.77 (C-40), 46.55 (C-21), 46.40 (C-22), 44.07 (C-4), 42.60 (C-7), 40.94 (C-1), 39.82 (C-8), 39.43 (C-10), 39.27 (C-3), 37.50 (C-12), 29.31 (C-24), 27.87 (C-20), 22.77 (C-6), 20.56 (C-11), 20.27 (C-2), 20.02 (C-17), 15.36 (C-19), 12.87 (C-41) ppm; MS (ESI, MeOH/CHCl_3_, 4:1): *m/z* (%) = 825 (92%, [M-Cl]^+^); analysis calcd for C_53_H_69_N_4_O_4_Cl (861.61): C 73.88, H 8.07, N 6.50; found: C 73.68, H 8.21, N 6.36.

### 3.36. 18-(4-[3-(2,3,6,7,12,13,16,17-Octahydro-1H,5H,11H,15H-pyrido[3,2,1-ij]-pyrido[1”,2”,3”:1′,8′]-quinolino[6′,5′:5,6]-pyrano[2,3-f]-quinoline-4-ium-9-yl)-benzoyl]-piperazin-1-yl)-18-oxostachan-16-one Chloride (33)

Following GPC from **24** (500 mg, 1.29 mmol), oxalyl chloride (0.6 mL, 6.78 mmol), rhodamine 101 (423.2 mg, 0.86 mmol), NEt_3_ (0.5 mL, 3.39 mmol), and chromatography (SiO_2_, CHCl_3_/MeOH, 95:5), **33** (416 mg, 36%) was obtained as a purple solid; R_f_ = 0.26 (SiO_2_, CHCl_3_/MeOH, 9:1); m.p. = 197 °C; UV-Vis (MeOH): λ_max_ (log ε) = 582.71 nm (1.14); IR (ATR): ν = 2845*w*, 1732*w*, 1633*w*, 1594*m*, 1542*w*, 1493*m*, 1458*w*, 1360*w*, 1293*s*, 1180*m*, 1087*s*, 1024*m*, 1002*m*, 747*w*, 621*m*, 420*m* cm^−1^; ^1^H NMR (500 MHz, DMSO-d_6_): δ = 7.78 − 7.53 (*m*, 6H, 28-H, 29-H, 30-H, 31-H, 35-H, 38-H), 7.40 (*dd*, *J* = 5.8, 2.7 Hz, 2H, 37-H), 6.66 (*d*, *J* = 2.8 Hz, 2H, 35-H), 3.50 (*dt*, *J* = 17.1, 5.6 Hz, 16H, 21-H, 22-H, 23-H, 24-H, 39-H), 3.04 − 2.91 (*m*, 10H, 40-H, 41-H), 2.66 (*t*, *J* = 6.5 Hz, 1H, 15-H), 2.26 − 2.15 (*m*, 1H, 3-H), 2.05 − 1.92 (*m*, 1H, 6-H), 1.85 (*q*, *J* = 9.3, 5.2 Hz, 2H, 6-H, 15-H), 1.76 − 1.54 (*m*, 4H, 1-H, 7-H, 11-H, 12-H), 1.54 − 1.29 (*m*, 12H, 2-H, 3-H, 7-H, 9-H, 11-H, 12-H, 14-H, 20-H), 1.23 (*d*, *J* = 2.9 Hz, 1H, 5-H), 1.18 (*s*, 3H, 20-H), 1.12 -0.95 (*m*, 3H, 17-H), 0.87 (*s*, 1H, 1-H), 0.69 (*s*, 3H, 19-H); ^13^C NMR (126 MHz, CDCl_3_): δ = 222.78 (C-16), 177.14 (C-18), 167.87 (C-25), 153.17 (C-36), 152.01 (C-32), 151.30 (C-34), 134.84 (C-27), 131.75 (C-26), 130.16 (C-28), 127.67 (C-38), 126.75 (C-33), 123.64 (C-30), 113.67 (C-37), 105.35 (C-35), 61.66 (C-5), 55.87 (C-9), 54.37 (C-14), 51.15 (C-13), 50.63 (C-15), 48.70 (C-21, C-24), 48.55 (C-22, C-23), 46.18 (C-4), 42.35 (C-39), 40.68 (C-7), 39.66 (C-1), 38.55 (C-8), 37.33 (C-3), 29.66 (C-10), 28.01 (C-12), 27.75 (C-20), 22.47 (C-6), 20.72 (C-41), 20.39 (C-40), 20.00 (C-11), 19.86 (C-17), 19.74 (C-2), 16.02 (C-19) ppm; MS (ESI, MeOH/CHCl_3_, 4:1): *m/z* (%) = 860 (90%, [M-Cl]^+^); analysis calcd for C_56_H_67_N_4_O_4_Cl (895.63): C 75.10, H 7.54, N 6.26; found: C 74.88, H 7.78, N 6.17.

### 3.37. 18-(4-[3-(2,3,6,7,12,13,16,17-Octahydro-1H,5H,11H,15H-pyrido[3,2,1-ij]-pyrido[1”,2”,3”:1′,8′]-quinolino[6′,5′:5,6]-pyrano[2,3-f]-quinoline-4-ium-9-yl)-benzoyl]-1,4-diazepan-1-yl)-18-oxostachan-16-one chloride (34)

Following GPZ from **25** (636 mg, 1.58 mmol), oxalyl chloride (0.6 mL, 6.78 mmol), rhodamine 101 (408 mg, 0.83 mmol), NEt_3_ (0.5 mL, 3.39 mmol), and chromatography (SiO_2_, CHCl_3_/MeOH, 95:5), **34** (532 mg, 37%) was obtained as a purple solid; R_f_ = 0.23 (SiO_2_, CHCl_3_/MeOH, 9:1); m.p. = 189 °C; UV-Vis (MeOH): λ_max_ (log ε) = 583.47 nm (1.79); IR (ATR): ν = 2924*s*, 1732*w*, 1594*m*, 1543*w*, 1493*m*, 1458*w*, 1361*w*, 1293*s*, 1181*m*, 1088*s*, 1025*m*, 746*m*, 621*m*, 421*m* cm^−1^; ^1^H NMR (500 MHz, DMSO-d_6_): δ = 7.75 − 7.61 (*m*, 6H, 29-H, 30-H, 31-H, 32-H, 39-H), 6.73 − 6.60 (*m*, 4H, 38-H, 39-H), 3.65 − 3.38 (*m*, 16H, 21-H, 22-H, 23-H, 24-H, 40-H), 3.26 (*d*, *J* = 21.3 Hz, 4H, 41-H, 42-H), 3.01 − 2.83 (*m*, 4H, 41-H, 42-H), 2.74 − 2.57 (*m*, 1H, 15-H), 2.48 − 2.40 (*m*, 1H, 6-H), 2.21 (*m*, 1H, 3-H), 1.90 − 1.76 (*m*, 4H, 6-H, 15-H, 24-H), 1.61 (*m*, 4H, 1-H, 7-H, 11-H, 12-H), 1.35 (*dtd*, *J* = 21.8, 12.8, 11.1, 6.9 Hz, 3H, 11-H, 12-H, 14-H), 1.25 − 1.18 (*m*, 3H, 2-H, 3-H, 7-H), 1.18 − 0.96 (*m*, 3H, 5-H, 7-H, 9-H), 0.94 (*d*, *J* = 12.8 Hz, 3H, 20-H), 0.87 (*d*, *J* = 5.5 Hz, 3H, 17-H), 0.75 (*s*, 1H, 1-H), 0.66 (*s*, 3H, 19-H) ppm; ^13^C NMR (126 MHz, CDCl_3_): δ = 222.81 (C-16), 176,39 (C-18), 168.42 (C-26), 163.05 (C-37), 152.01 (C-33), 152.94 (C-35), 151.35 (C-39), 131.33 (C-28), 130.69 (C-27), 130.15 (C-29), 129.76 (C-30), 129.21 (C-32), 127.75 (C-31), 123.84 (C-38), 123.43 (C-34), 113.27 (C-36), 62.49 (C-5), 62.09 (C-9), 56.07 (C-14), 54.44 (C-41), 50.94 (C-25), 49.97 (C-23), 48.72 (C-13), 48.67 (C-15), 47.14 (C-40), 46.73 (C-21), 46.58 (C-22), 46.37 (C-42), 44.92 (C-4), 42.57 (C-7), 40.98 (C-1), 40.81 (C-8), 39.71 (C-10), 38.64 (C-3), 37.34 (C-12), 28.51 (C-24), 27.54 (C-20), 22.64 (C-6), 20.61 (C-11), 19.90 (C-2), 19.67 (C-17), 16.27 (C-19) ppm; MS (ESI, MeOH/CHCl_3_, 4:1): *m/z* (%) 874 (96%, [M-Cl]^+^); analysis calcd for C_57_H_69_N_4_O_4_Cl (909.64): C 75.26, H 7.65, N 6.16; found: C 75.01, H 7.83, N 5.97.

## 4. Conclusions

Acid hydrolysis of stevioside resulted in a 63% yield of isosteviol (1), which was used as a starting material for the preparation of numerous amides. These amides were tested for cytotoxic activity; while almost all the amides were found to be non-cytotoxic in a panel of human tumor cell lines, only the combination of isosteviol, a (homo)piperazinyl spacer and rhodamine B or rhodamine 101 unit proved to be particularly suitable. These spacered rhodamine conjugates showed cytotoxic activity in the sub-micromolar concentration range. In this regard, the homopiperazinyl-spacered derivatives were found to be better than the piperazinyl-spacered compounds, and the rhodamine 101 conjugates were more cytotoxic than the rhodamine B.

## Figures and Tables

**Table 1:** Cytotoxicity of compounds: isosteviol (1) and compounds **2**–**34** (EC_50_-values in µM from SRB-assays) after 72 h of treatment; the values are averaged from three independent experiments performed each in triplicate, confidence interval CI = 95%; mean ± standard mean error; malignant cell lines tested: A375 (melanoma), HT29 (colon adenocarcinoma), MCF-7 (breast adenocarcinoma), A2780 (ovarian carcinoma), HeLa (cervical adenocarcinoma); non-malignant cell lines tested: NIH 3T3 (murine fibroblast), HEK293 (embryonic kidney); positive control: doxorubicin (DOX); n.d. not determined.

	A375	HT29	MCF7	A2780	HeLa	NIH 3T3	HEK293
**1**	>20	>20	>20	>20	>20	>20	>20
**2–23**	>20	>20	>20	>20	>20	>20	>20
**24**	>20	13.5± 2.1	>20	>20	>20	11.2 ± 1.1	>20
**25**	14.3 ± 1.7	11.2 ± 1.3	11.1 ± 0.9	13.0 ± 1.3	>20	11.9 ± 2.50	>20
**26–30**	>20	>20	>20	>20	>20	>20	>20
**31**	0.91 ± 0.02	0.81 ± 0.04	0.42 ± 0.04	0.40 ± 0.02	0.81 ± 0.03	1.28 ± 0.11	0.40 ± 0.05
**32**	0.94 ± 0.15	0.90 ± 0.06	0.36 ± 0.06	0.29 ± 0.08	1.34 ± 0.9	1.33 ± 0.09	0.63 ± 0.07
**33**	0.44 ± 0.01	0.42 ± 0.04	0.23 ± 0.02	0.19 ± 0.01	0.45 ± 0.06	0.59 ± 0.02	0.20 ± 0.02
**34**	0.40 ± 0.02	0.47 ± 0.09	0.23 ± 0.02	0.16 ± 0.02	0.44 ± 0.07	0.08 ± 0.07	0.25 ± 0.04
**DOX**	n.d.	0.91 ± 0.01	1.10 ± 0.30	0.01 ± 0.01	n.d.	0.41 ± 0.07	n.d.

## Data Availability

The data presented in this study are available on request from the corresponding author.

## Sample Availability

Samples of the compounds are available from the authors.
